# Supplementary material for: Social media use and adolescent sleep patterns: cross-sectional findings from the UK millennium cohort study
Source: BMJ Open. 2019 Oct 22;9(9):e031161. doi: 10.1136/bmjopen-2019-031161 (PMC6830469; doi:10.1136/bmjopen-2019-031161)
Supplement: Supplementary data [file bmjopen-2019-031161supp001.pdf]

Supplementary Materials

Full breakdown of sleep patterns

Supplementary Table 1. Typical sleep onset (school days) for males & females

Typical sleep onset on school days	Male		Female		Total	
	%	n	%	n	%	n
Before 9 pm	5.8	288	4.7	248	5.2	536
9 - 9:59 pm	28.5	1619	30.0	1681	29.2	3300
10 - 10:59 pm	40.2	2352	38.8	2284	39.5	4636
11 pm - midnight	18.7	1076	20.3	1220	19.5	2296
After midnight	6.9	366	6.1	335	6.5	701

Notes: percentages account for survey design and weights.

Supplementary Table 2. Typical sleep onset (free days) for males & females

Typical sleep onset on free days	Male		Female		Total	
	%	n	%	n	%	n
Before 9 pm	0.9	53	0.9	45	0.9	98
9 - 9:59 pm	6.0	328	5.4	299	5.7	627
10 - 10:59 pm	22.8	1311	24.4	1407	23.6	2718
11 pm - midnight	35.1	2044	37.2	2208	36.1	4252
After midnight	35.2	1969	32.1	1808	33.7	3777

Notes: percentages account for survey design and weights. Gender difference in sleep onset (free days) $p < .05$.

Supplementary Table 3. Typical wake time (school days) for males & females

Typical wake time on school days	Male		Female		Total	
	%	n	%	n	%	n
Before 6 am	4.4	220	4.5	230	4.5	450
6 - 6:59 am	37.0	1995	47.9	2609	42.2	4604
7 - 7:59 am	53.3	3205	44.9	2771	49.3	5976
8 - 8:59 am	4.0	247	2.1	130	3.1	377
After 9 am	1.3	42	0.6	32	1.0	74

Notes: percentages account for survey design and weights. Gender difference in wake time (school days) $p < .001$.

Supplementary Table 4. Typical wake time (free days) for males & females

Typical wake time on free days	Male		Female		Total	
	%	n	%	n	%	n
Before 8 am	9.9	545	6.4	342	8.2	887
8 - 8:59 am	17.1	940	15.6	846	16.3	1786
9 - 9:59 am	22.9	1348	26.5	1537	24.6	2885
10 - 10:59 am	27.6	1628	30.2	1763	28.8	3391
11 - 11:59 am	14.8	836	15.3	934	15.0	1770
After midday	7.8	401	6.2	348	7.0	749

Notes: percentages account for survey design and weights. Gender difference in wake time (free days) $p < .001$.

Supplementary Table 5. Typical sleep onset latency for males & females

Typical sleep onset latency	Male		Female		Total	
	%	n	%	n	%	n
0 - 15 minutes	37.1	2104	29.3	1709	33.3	3813
16 - 30 minutes	31.4	1838	33.7	1963	32.6	3801
31 - 45 minutes	14.8	819	17.3	973	16.0	1792
46 - 60 minutes	6.4	383	8.7	495	7.5	878
More than 60 minutes	10.3	516	11.0	610	10.6	1126

Notes: percentages account for survey design and weights. Gender difference in sleep onset latency $p < .001$.

Supplementary Table 6. Frequency of nighttime awakenings for males & females

Frequency of nighttime awakenings	Male		Female		Total	
	%	n	%	n	%	n
None of the time	35.8	2050	24.1	1435	30.1	3485
A little of the time	32.8	1951	32.7	1966	32.8	3917
Some of the time	14.5	831	17.6	991	16.0	1822
A good bit of the time	7.0	380	10.7	583	8.8	963
Most of the time	6.7	337	10.7	554	8.6	891
All of the time	3.1	143	4.3	226	3.7	369

Notes: percentages account for survey design and weights. Gender difference in nighttime awakenings $p < .001$.

Social media use by demographics

Table 1 in the main text provides a breakdown of social media use by gender. Supplementary tables 7 and 8 provide a breakdown of social media use by additional demographics: household income and ethnic minority status.

Supplementary Table 7. Social media use by household equivalised income

		Social media use				
		Low<br><1 h	Average<br>1 to <3 h	High<br>3 to <5 h	Very high<br>5+ h	**Total n**
**Household equivalized income**	Quintile 1 (lowest)	33.9	28.7	11.3	26.1	2038
	Quintile 2	30.6	29.2	16.3	23.9	2008
	Quintile 3	31.6	30.3	15.2	22.9	2414
	Quintile 4	32.4	35.8	14.0	17.9	2727
	Quintile 5 (highest)	40.0	33.8	12.7	13.6	2685

Notes: Numbers represent row percentages (e.g. 33.9% of cohort members in lowest income quintile were low social media users). Percentages account for survey design and weights. Income difference in social media use $p < .001$.

Supplementary Table 8. Social media use by ethnicity

		Social media use				
		Low<br><1 h	Average<br>1 to <3 h	High<br>3 to <5 h	Very high<br>5+ h	**Total n**
**Ethnicity**	White	32.9	31.7	14.4	21.1	9086
	Non-white	37.3	31.4	11.9	19.4	2326

Notes: Numbers represent row percentages (e.g. 32.9% of White cohort members were low social media users). Percentages account for survey design and weights. Ethnicity difference in social media use $p < .05$.

Survey questions

About what time do you usually **go to sleep** on a **school night**?
1 Before 9 pm
2 9 - 9:59 pm
3 10 – 10:59 pm
4 11 - midnight
5 After midnight

About what time do you usually **wake up** in the morning on a **school day**?
1 Before 6 am
2 6 - 6:59 am
3 7 – 7:59 am
4 8 - 8:59 am
5 After 9 am

About what time do you usually **go to sleep** on the nights when you **do not have school** the next day?
1 Before 9 pm
2 9 - 9:59 pm
3 10 - 10:59 pm
4 11 - midnight
5 After midnight

About what time do you **wake up** in the morning on the days when you **do not have school**?
1 Before 8 am
2 8 - 8:59 am
3 9 – 9:59 am
4 10 - 10:59 am
5 11 - 11:59 am
6 After Midday

During the last four weeks, how long did it usually take for you to fall asleep?
1 0-15 minutes
2 16-30 minutes
3 31-45 minutes
4 46-60 minutes
5 More than 60 minutes

During the last four weeks, how often did you awaken during your sleep time and have trouble falling back to sleep again?
1 All of the time
2 Most of the time
3 A good bit of the time
4 Some of the time
5 A little of the time
6 None of the time

On a normal week day during term time, how many hours do you spend on social networking or messaging sites or Apps on the internet such as Facebook, Twitter and WhatsApp?
1 None
2 Less than half an hour
3 Half an hour to less than 1 hour
4 1 hour to less than 2 hours
5 2 hours to less than 3 hours
6 3 hours to less than 5 hours

7 5 hours to less than 7 hours
8 7 hours or more

Figures

Figure 1. Odds Ratios of sleep outcomes for low, high & very high daily social media users

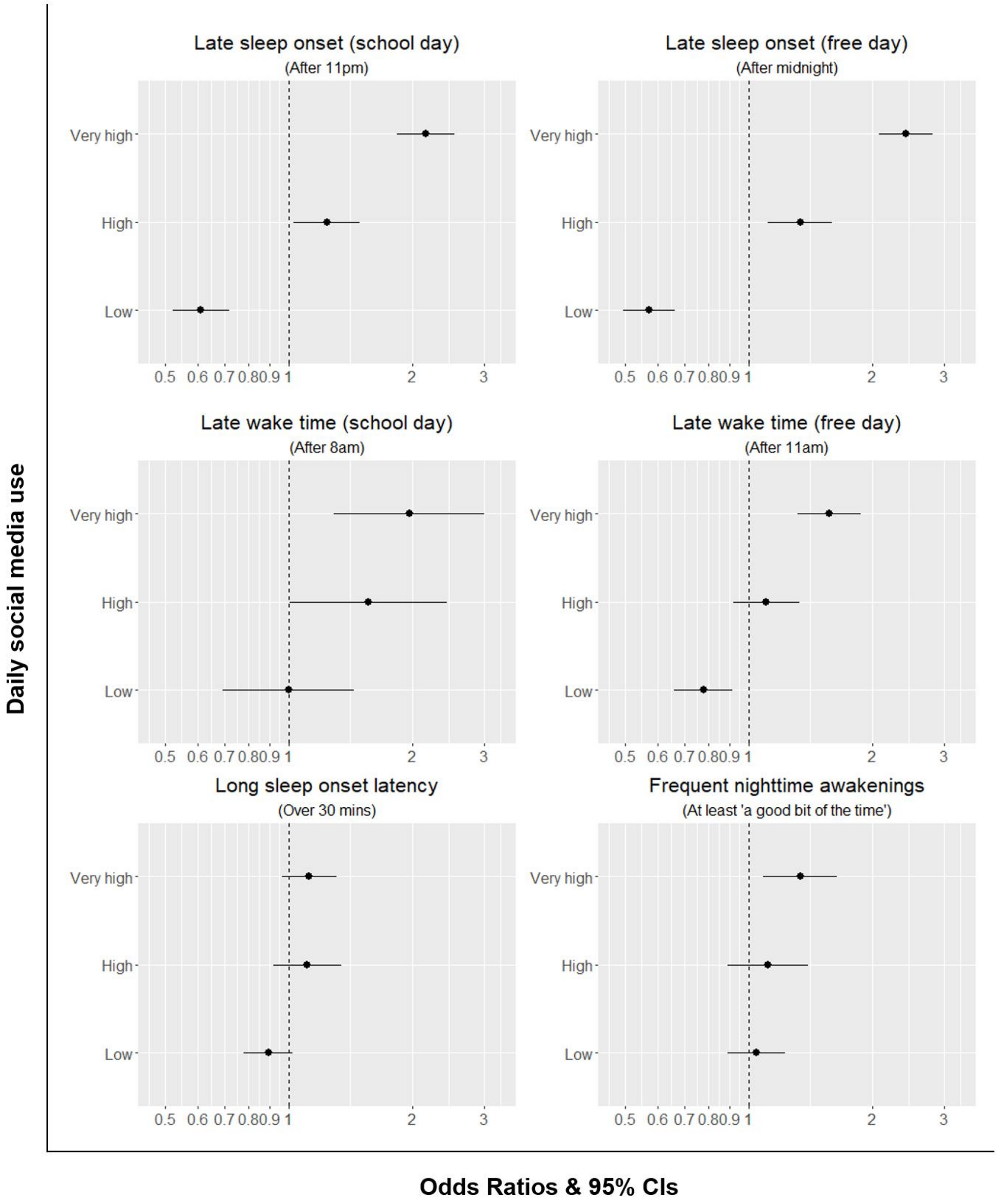

Notes: Odds Ratios measure how much higher or lower the odds of a given sleep outcome are for each category of social media user (Low, <1h; High, 3-5h; Very high, 5+h) compared to typical users (1-3h). Odds Ratios and 95% Confidence Intervals greater than 1 indicate higher odds; those below 1 indicate lower odds. Odds Ratios are adjusted to control for: exact age, sex, ethnic minority status, family income, number of siblings in household, presence of both parents, parent age, Strengths and Difficulties score, Mood and Feelings score, self-esteem, general health, social support and physical activity.
