# Supplementary material for: Social media use and adolescent sleep patterns: cross-sectional findings from the UK millennium cohort study
Source: BMJ Open. 2019 Oct 22;9(9):e031161. doi: 10.1136/bmjopen-2019-031161 (PMC6830469; doi:10.1136/bmjopen-2019-031161)
Supplement: Author's manuscript [file bmjopen-2019-031161.draft_revisions.pdf]

BMJ Open is committed to open peer review. As part of this commitment we make the peer review history of every article we publish publicly available.

When an article is published we post the peer reviewers' comments and the authors' responses online. We also post the versions of the paper that were used during peer review. These are the versions that the peer review comments apply to.

The versions of the paper that follow are the versions that were submitted during the peer review process. They are not the versions of record or the final published versions. They should not be cited or distributed as the published version of this manuscript.

BMJ Open is an open access journal and the full, final, typeset and author-corrected version of record of the manuscript is available on our site with no access controls, subscription charges or pay-per-view fees (http://bmjopen.bmj.com).

If you have any questions on BMJ Open's open peer review process please email info.bmjopen@bmj.com

BMJ Open

Social media use and adolescent sleep outcomes: cross-sectional findings from the UK Millennium Cohort Study

Journal:	*BMJ Open*
Manuscript ID	bmjopen-2019-031161
Article Type:	Research
Date Submitted by the Author:	23-Apr-2019
Complete List of Authors:	Scott, Holly; University of Glasgow, School of Psychology<br>Biello, Stephany; University of Glasgow, School of Psychology<br>Woods, HC; University of Glasgow, School of Psychology
Keywords:	Social media use, Sleep, Adolescents, Delayed bedtime, Sleep quality, Screen time

SCHOLARONE™
Manuscripts

TITLE PAGE

Title

Social media use and adolescent sleep patterns: cross-sectional findings from the UK Millennium Cohort Study

Author names

Holly Scott, MSc; Stephany M Biello, PhD; Heather Cleland Woods, PhD

Author job titles & affiliation address

Holly Scott, PhD candidate

Stephany M Biello, Professor & Depute Head of School

Heather Cleland Woods, Lecturer

All authors have same institutional affiliation/address:

School of Psychology, University of Glasgow, 62 Hillhead Street, Glasgow, G12 8QB.

Corresponding author

Holly Scott, h.scott.1@research.gla.ac.uk, postal address as above.

For peer review only

ABSTRACT

Objectives

This study examines associations between social media use and multiple sleep parameters in a large representative adolescent sample. It aims to establish a normative profile of social media use amongst UK adolescents, and quantify associated sleep patterns for higher users relative to average users, controlling for a wide range of covariates.

Design

The authors used cross-sectional data from the Millennium Cohort Study, a large nationally representative UK birth cohort study.

Participants

Data from 11,872 adolescents (aged 13-15 years) were used in analyses.

Methods

Six self-reported sleep parameters captured both sleep timing and quality: sleep onset and wake times (on school days and free days), sleep onset latency (time taken to fall asleep) and trouble falling back asleep after nighttime awakening. Binomial logistic regressions investigated associations between daily social media use and each sleep parameter, controlling for a range of relevant sociodemographic, health and lifestyle covariates.

Results

Overall, heavier social media use was associated with poorer sleep patterns, controlling for covariates. Very high social media users (at least 5h per day, 20.8%) were more likely than comparable average users (1 to <3h per day, 31.6%) to report late sleep onset (OR = 2.14, 95% CI: 1.83 to 2.50) and wake times (OR = 1.97, 95% CI: 1.32 to 2.93) on school days, and trouble falling back asleep after nighttime awakening (OR = 1.36, 95% CI: 1.10 to 1.66).

Conclusions

Results indicate statistically and practically significant associations between social media use and sleep patterns, particularly late sleep onset. Interventions can focus on supporting young people to balance online interactions with an appropriate sleep schedule for sufficient sleep on school nights. Future research should explore the context and experience of social media use, to inform meaningful discussions on updating sleep education and interventions to meet the needs of today’s society.

For peer review only

STRENGTHS AND LIMITATIONS

- Provides a current normative profile of social media use and sleep in UK adolescents
- Moves beyond generic “screen time” to examine social media specifically
- Uses data from a large representative sample, including comprehensive covariates
- Uses self-reported measures of social media use and sleep patterns
- Measures only duration of social media use, rather than content and context

For peer review only

INTRODUCTION

There is significant current attention towards the possible impact of screentime and social media on our adolescents' health. However, the lack of empirical evidence to support policy and practice development in this area has been consistently voiced by clinicians and researchers. For example, at the UK House of Commons Science and Technology Committee inquiry into the impact of social media and screen-use on young people's health use in adolescence, the Royal College of Paediatrics and Child Health (RCPCH) urged the UK government as a matter of priority to develop guidance for health practitioners along the same lines as the American Academy of Paediatrics (AAP) but importantly based on UK data[1 2]. They also argue along with other researchers that there is a need to refocus away from correlations between generic terms such as "screentime" and poor wellbeing, towards meaningfully quantifying how various types of technology use impact on different areas of child and adolescent health and wellbeing. This study presents UK data that provide a nationally representative profile of current adolescent social media use, and takes a data-driven approach to quantify sleep patterns for high and very high users relative to average users.

This study focuses on sleep, which – despite often being overlooked in public health messages and education interventions[3 4] – is increasingly recognised as a key component of wider health and wellbeing[5]. Adolescent sleep is an important public health issue, as insufficient sleep is highly prevalent in this age group and has implications for mental health, obesity, academic performance and safety[6]. With the majority of adolescents reporting insufficient sleep to function properly or to meet recommended guidelines[7 8], there is growing concern that social media may be a contributing factor for today's teenagers. For example, the potential for 24/7 social media interactions may exacerbate the existing conflict of early school start times with naturally delayed adolescent rhythms and other social and educational demands[6 9 10]. As a highly relevant issue for paediatric practice, there is a clear need for UK evidence to inform and update decision making in medical practice and policy to address this current issue in adolescent sleep.

This study responds to this need, presenting large-scale UK data on adolescent sleep and social media use, whilst addressing a number of existing gaps in available international

For peer review only

evidence. In addition to providing much-needed UK evidence, the current approach also addresses the need for evidence that: (1) examines social media specifically, rather than generic screentime; (2) isolates effects for a range of sleep parameters by accounting for an extensive range of covariates; (3) frames these effects within the context of current adolescent social media norms to provide meaningful comparisons. The current approach to ensure that this evidence can meaningfully inform policy and practice by addressing each of these needs is discussed further below.

Firstly, it is important for available evidence to examine social media individually, rather than aggregating these interactions and other media use under the umbrella term "screentime". A recent large-scale US study indicated a significant but modest effect for overall screentime and sleep, and called for future research to examine effects for specific technologies [11]. In particular, the interactive nature of social media presents uniquely relevant issues for adolescent sleep compared to other forms of screentime or traditional media [7 12]. Although facilitated by screens, social media interactions are underpinned by the same drivers as any social interaction, with a desire for inclusion and belonging mixing with concerns over violating social expectations or etiquette [13]. These concerns can make it difficult to disengage from social media at bedtime, with some adolescents identifying this as a cause of delayed sleep onset and daytime tiredness [13]. These unique social and emotional aspects of online interactions underline the importance of examining social media use specifically, in relation to adolescent sleep outcomes.

Secondly, to meaningfully inform an evidence-based response to social media use, research must examine multiple sleep parameters and isolate these effects by controlling for an extensive range of relevant covariates. This is crucial to assess the practical significance of underlying direct effects [11] and to identify which aspects of adolescent sleep merit attention to social media use in practice and policy. Available research on social media use and sleep is often left questioning whether reported effects could be explained by other individual factors: for example if more anxious, depressed or sedentary adolescents may tend to both use social media more and report poorer sleep [12 14]. Individual studies that have controlled for specific groups of covariates generally suggest that associations do persist [15 16]. However, there remains a need for large-scale evidence that addresses a wide range of covariates

For peer review only

simultaneously, to more robustly establish which dimensions of sleep have a direct association with social media use and which reflect another underlying issue (e.g. anxious or depressive symptoms).

This type of evidence is required to invest time and resources effectively, by identifying which sleep complaints may benefit from directly addressing social media use. For example, a range of sleep complaints from insufficient sleep to problems initiating or maintaining sleep have been examined in relation to social media use. In terms of sleep duration, time spent using social media may displace sleep directly or displace other daytime activities (such as homework) that are then delayed and disrupt nighttime routines [9 17]. Social media use may also impact on the quality of sleep via increased arousal, not simply though light exposure [18], but particularly via cognitive and social activity [12 14 19]. Given these different potential mechanisms, it is therefore important to examine social media use in relation to a range of sleep parameters, to identify which of these links have the most practical significance after accounting for relevant factors.

Thirdly, evidence should frame these effects within the context of current norms for adolescent social media use. Research to date has tended to focus on problematic or "addicted" social media users [10 20], or to compare outcomes for the highest users against the lowest users [15 17 21]. In contrast, first establishing what constitutes typical use and then comparing outcomes for relatively higher or lower users against this reference point can support more meaningful conclusions. This data-driven approach avoids imposing arbitrary or quickly outdated cut-offs, taking into account recent rapid increases in social media use [22]. Comparing sleep patterns for higher users against average users can better support practical and realistic discussions on best practice, that consider the context of current adolescent social media norms.

This study targets these existing gaps in available international evidence, while providing much-needed large-scale UK evidence. It examines associations between social media use and multiple sleep parameters in a large, nationally representative adolescent sample: the UK Millennium Cohort Study [23]. It first investigates current norms in adolescent social media use to establish the average level of daily use and the prevalence of comparatively high use.

For peer review only

It then examines which sleep parameters are associated with social media use, isolating these effects by controlling for extensive covariates and quantifying effects for higher users relative to average users. This aims to provide rigorous and meaningful evidence to inform practice and policy to support healthy adolescent sleep and social media use.

METHODS

Participants

The UK Millennium Cohort Study is a nationally representative, multidisciplinary survey which aims to explore the influence of family context on child and adolescent development and outcomes. The survey covers a broad range of domains, such as parenting, housing, poverty and health. It consists of a random 2-stage sample drawn from all live UK births in the 12-month period starting 1 September 2000 in England and Wales and 1 December 2000 in Scotland and Northern Ireland, identified through the Child Benefit register [23]. The clustered sample is drawn from a disproportionately stratified sample of electoral wards (local areas) to provide adequate representation of areas with higher concentrations of minority ethnic and disadvantaged families. Parents completed the first survey sweep in 2001 when their child was aged 9 months, with 18,818 cohort members. Children also completed surveys from age 7 (sweep 4) onwards. The most recent survey (sweep 6 at around age 14) gathered self-report data from 11,872 cohort members, including questions on their typical social media use and sleep patterns. Parents were required to give written consent to complete the parent survey and for the interviewer to invite the cohort member to participate in the young person survey. Cohort members then also had to give verbal consent to complete the young person survey, which was self-completed on the interviewer's tablet.

Materials

The current analyses make use of available data from the UK Millennium Cohort Study, which measured social media use and sleep using single-item self-report questions. Although not validated questionnaire measures, these survey questions do provide a snapshot of the subjective experience of sleep and social media use in this large representative sample, capturing a range of sleep habits and the typical time spent using social media each day.

For peer review only

Social media use

Participants indicated how much time they spent using social media on a typical weekday, choosing from 8 response categories (ranging from 0 hours to 7+ hours).

Sleep parameters

Participants reported typical sleep habits through six single items (each with 5 or 6 response categories) that assessed: sleep onset and wake times (on school days and free days, separately), sleep onset latency (time taken to fall asleep) and trouble falling back asleep after nighttime awakening. The Supplementary Materials provide a full list of items and response categories.

Covariates

In addition, the following relevant covariates (identified based on literature) had available data in the UK Millennium Cohort Study: demographics (ethnic minority status, OECD equivalised weekly family income); family composition (number of siblings in household, presence of both parents, age of primary parent/carer responder); psychosocial adjustment (using the parent-report Strengths and Difficulties Questionnaire)[24]; depressive symptoms (using the Short Mood and Feelings Questionnaire)[25]; self-esteem (using a shortened and adapted version of the Rosenberg Self-Esteem Scale)[26]; general health (single item), social support (three items) and physical activity (single item).

Data analysis

Since the aim of the study was to compare sleep outcomes for high and low users versus average users, based on the distribution we initially collapsed responses into three categories: under 1 hour for 'low' users (33.7%), 1 to <3 hours for 'average' users (31.6%), and 3 hours or more for 'high' users (34.7%). Given the broad range covered by this 'high' user category (including responses of 3 to <5, 5 to <7 and 7+ hours), and with sufficient numbers, we separated this into 'high' (3 to <5 hours; 13.9%) and 'very high' users (5+ hours; 20.8%) to allow more detailed exploration.

We collapsed responses for each sleep measure into binary outcomes. For poor sleep quality, these outcomes were: sleep onset latency over 30 minutes [27] and difficulty falling

For peer review only

asleep following nighttime awakenings at least 'a good bit of the time'. For late sleep onset and wake times, we took a data-driven approach to identify meaningful cut-off points, which were defined as later than average (including responses in categories later than the median response category). Table 1 summarises the resulting criteria for each sleep outcome and associated prevalence rates.

Table 1 Social media use and sleep outcomes: criteria and prevalence

Variable	Criteria	Prevalence (%)		
		Male	Female	Total
Daily social media use				
Low	< 1 hour	43.8	22.8	33.7
Average	1 to <3 hours	32.1	31.1	31.6
High	3 to <5 hours	10.4	17.7	13.9
Very high	5+ hours	13.7	28.4	20.8
Sleep outcomes				
Late sleep onset (school day)	After 11pm	25.5	26.5	26.0
Late sleep onset (free day)	After midnight	35.2	32.1	33.7
Late wake time (school day)	After 8am	5.3	2.7	4.0
Late wake time (free day)	After 11am	22.5	21.5	22.0
Long sleep onset latency	Over 30 minutes	31.5	37.0	34.1
Trouble falling back asleep after nighttime awakening	At least 'a good bit of the time'	16.9	25.7	21.1

Notes: Percentages account for survey design and weights. Criteria for late sleep onset and wake times defined as later than the median response category. Gender difference in daily social media use, late wake time (school day), long sleep onset latency and trouble falling back asleep after nighttime awakening $p < .001$; gender difference in late sleep onset (free day) $p < .01$. For a breakdown of social media use by other demographics (household income and ethnicity), see the Supplementary Materials.

Separate binomial logistic regression models predicted Odds Ratios of each sleep outcome for low, high and very high social media users, compared to average users. We ran models that controlled only for exact age and sex, followed by models that further controlled for measures of demographics, family characteristics, psychological wellbeing and health (see Materials section for full details). All analyses allowed for the complex survey design (with its clustered, stratified sample) and used longitudinal weights to account for non-random longitudinal attrition from the sample, using the 'survey' and 'srvyr' packages in R [28-30].

For peer review only

Multiple imputation was performed to account for missing data, reducing bias and increasing power [31]. The overall missing data rate was 2.8%, ranging from 0.0% to 6.0% for individual measures, with most measures below 5%. We make the assumption that data are missing at random (i.e. that patterns of missingness can be explained by other variables available in the data; [31]). All variables, including covariates, were used in the imputation model, which was run using R package 'mice' [32]. Estimates were combined across 10 imputed data sets (each produced through 10 iterations). Results were similar for analyses on multiply imputed and complete case data, so only multiply imputed analysis is presented here.

RESULTS

The median time spent using social media on a typical day was 1 to <3 hours (32% of adolescents); however, 21% used social media for at least 5 hours. Girls tended to use social media more than boys (see Table 1).

Median sleep onset times were 10-11pm on school days (with 26% falling asleep later than this) and between 11pm and midnight on free days (with 34% falling asleep later; see Table 1). Median wake times were 7-8am on school days (with only 4% waking later than this) and 10-11am on free days (with 22% waking later). Boys were more likely to fall asleep late on free days and wake up late on school days. In measures of poor sleep quality, 34% typically took longer than 30 minutes to fall asleep and 21% reported difficulties falling asleep following nighttime awakenings at least 'a good bit of the time'. Girls were more likely to have long sleep onset latency and trouble falling back asleep after nighttime awakening.

Separate binomial logistic regression models explored whether odds of each sleep outcome differed for low, high and very high social media users, compared to average users (1 to <3 h). First, models controlled for exact age and sex (see Table 2). Very high social media use (5+ h) was associated with higher odds of all six sleep outcomes. High social media use (3 to <5 h) was associated with higher odds of all outcomes except for late rise times on free days. Low social media use (<1 h) was associated with lower odds of late sleep onset on school- and free- days, and late wake times on free days.

For peer review only - http://bmjopen.bmj.com/site/about/guidelines.xhtml

Table 2 Binomial logistic regressions (adjusting only for age & sex)

	Low: <1 h OR (95% CI)	Average: 1 to <3 h	High: 3 to <5 h OR (95% CI)	Very high: 5+ h OR (95% CI)
Late sleep onset (school days)	0.63*** (0.53, 0.75)	Reference group	1.38*** (1.16, 1.65)	2.75*** (2.38, 3.18)
Late sleep onset (free days)	0.6*** (0.51, 0.7)	"	1.44*** (1.19, 1.74)	3.05*** (2.63, 3.54)
Late wake time (school days)	1.12 (0.77, 1.62)	"	1.63* (1.05, 2.53)	2.49*** (1.62, 3.83)
Late wake time (free days)	0.81* (0.69, 0.96)	"	1.16 (0.97, 1.39)	1.82*** (1.54, 2.16)
Sleep Onset Latency > 30 mins	0.92 (0.8, 1.05)	"	1.24* (1.04, 1.49)	1.48*** (1.27, 1.71)
Frequent nighttime awakenings	1.06 (0.9, 1.26)	"	1.31* (1.07, 1.61)	2.11*** (1.75, 2.55)

Notes: See Table 2 for criteria and prevalence of each sleep outcome. **p* < .05, ***p* < .01, ****p* < .001. Odds Ratios measure how much higher or lower the odds of a given sleep outcome are for each category of social media user (Low, <1h; High, 3 to <5h; Very high, 5+h) compared to average users (1 to <3h). Odds Ratios and 95% Confidence Intervals greater than 1 indicate higher odds; those below 1 indicate lower odds. Odds Ratios are adjusted to control for exact age & sex.

Further modelling then controlled for a more comprehensive set of covariates (see Table 3, with note detailing list of covariates). High social media use was no longer significantly associated with long sleep onset latency or frequent nighttime awakenings. Very high social media was no longer significantly associated with long sleep onset latency; however, its association with frequent nighttime awakenings remained significant but smaller. Patterns of significant associations for late sleep onset and wake times remained unchanged, although effect sizes were reduced, particularly for very high social media use.

Table 3 Binomial logistic regressions (with further adjustments for covariates)

	Low: <1 h OR (95% CI)	Average: 1 to <3 h	High: 3 to <5 h OR (95% CI)	Very high: 5+ h OR (95% CI)

For peer review only

Late sleep onset (school days)	0.61*** (0.51, 0.73)	Reference group	1.23* (1.02, 1.49)	2.14*** (1.83, 2.5)
Late sleep onset (free days)	0.57*** (0.49, 0.68)	"	1.32** (1.09, 1.6)	2.41*** (2.08, 2.79)
Late wake time (school days)	1.04 (0.71, 1.5)	"	1.56* (1.02, 2.4)	1.97** (1.32, 2.93)
Late wake time (free days)	0.79** (0.67, 0.93)	"	1.12 (0.92, 1.35)	1.57*** (1.32, 1.87)
Sleep Onset Latency > 30 mins	0.9 (0.78, 1.04)	"	1.11 (0.92, 1.34)	1.12 (0.96, 1.32)
Frequent nighttime awakenings	1.04 (0.88, 1.24)	"	1.08 (0.87, 1.35)	1.36** (1.1, 1.66)

Notes: See Table 2 for criteria and prevalence of each sleep outcome. **p* < .05, ***p* < .01, ****p* < .001. Odds Ratios are adjusted to control for: exact age, sex, ethnic minority status, family income, number of siblings in household, presence of both parents in household, parent age, Strengths and Difficulties score, Mood and Feelings score, self-esteem, general health, social support and physical activity.

For ease of interpretation, we also transformed the resulting adjusted Odds Ratios from these covariate models into adjusted Relative Risks [33] (see Table 4). These summarise differences in *probabilities,* as opposed to odds, and can be interpreted more intuitively. For example, the adjusted relative risk of 1.68 indicates that an adolescent with very high social media use is 68% more likely to fall asleep after 11pm on school nights than a comparable adolescent (controlling for covariates) with average social media use.

Table 4 Relative Risks (from covariate-adjusted models)

	Low: <1 h RR (95% CI)	Average: 1 to <3 h	High: 3 to <5 h RR (95% CI)	Very high: 5+ h RR (95% CI)
Late sleep onset (school days)	0.67*** (0.58, 0.78)	Reference group	1.17* (1.02, 1.33)	1.68*** (1.52, 1.84)
Late sleep onset (free days)	0.66*** (0.58, 0.75)	"	1.2** (1.06, 1.35)	1.69*** (1.57, 1.81)

For peer review only

Late wake time (school days)	1.03 (0.72, 1.48)	“	1.54* (1.02, 2.29)	1.91** (1.3, 2.76)
Late wake time (free days)	0.83** (0.72, 0.95)	“	1.09 (0.94, 1.26)	1.41*** (1.24, 1.59)
Sleep Onset Latency > 30 mins	0.93 (0.84, 1.03)	“	1.07 (0.94, 1.21)	1.08 (0.97, 1.2)
Frequent nighttime awakenings	1.03 (0.89, 1.19)	“	1.07 (0.89, 1.28)	1.28** (1.09, 1.5)

Notes: Relative Risks transformed from Odds Ratios in covariate-controlled binomial logistic regression models (see Table 3). E.g. RR of 1.68 means very high users are 68% more likely to fall asleep late on school days than comparable average users. *$p < .05$, **$p < .01$, ***$p < .001$.

For peer review only

DISCUSSION

This study aimed to address calls from those working in policy and practice to establish a UK data driven profile of current adolescent daily social media use, and to examine links to a key component of wider adolescent health and wellbeing using multiple sleep parameters whilst accounting for a wide range of covariates, using data from a large nationally representative sample of UK adolescents. The results highlighted a wide range of reported daily social media use, with tertiles defining low, average and high use on a typical school day as <1 hour, 1 to <3 hours and 3+ hours respectively. This indicates generally heavier social media use compared to young adults [21] and provides a current normative profile for UK adolescents. One in five adolescents were classed as very high users, spending 5+ hours using social media on a typical school day, whereas two thirds of the sample used social media for less than 3 hours. This provides a data-driven profile of use to support decision-making, rather than relying on assumptions around prevalence of high use. In line with previous studies, girls tended to spend more time on social media than boys[34 35] and report poorer sleep quality [36 37]. This reinforces the importance of controlling for gender when examining these associations, and highlights the need for continued work to explore the sleep implications of *how* adolescent boys and girls spend their time on social media (with previous evidence of gender differences in preferred platforms, motivations and self-presentation [34 35 38]).

In terms of sleep timing, social media use remained significantly associated with late sleep onset and wake times after controlling for covariates, with the strongest effect for sleep onset. Very high social media users were roughly 70% more likely than comparable average users to fall asleep later than average, i.e. after 11pm on school days and after midnight on free days. Low social media users were least likely to fall asleep late, indicating that unlike mental wellbeing, optimal outcomes for sleep are associated with minimal – not moderate – use [39]. These findings are consistent with the idea that social media displaces sleep: either directly or indirectly [9 17]. Direct sleep displacement may be particularly likely on school days, especially for very high users, since limited social media access during school hours means that at least part of this daily time on social media is likely to take place close to bedtime. Bedtime social media use can delay sleep onset [14], with some adolescents reporting difficulties disengaging from social media to sleep [13]. A similar process could also indirectly delay sleep onset, if other daytime activities (e.g. homework) are delayed due to a sense of urgency to check and respond to social media notifications. This link to later sleep onset is a particular concern on school days, as late school day bedtimes longitudinally predict poorer academic and emotional outcomes [40]. Whilst the survey question aimed to measure sleep onset time by asking what time participants "go to sleep", some participants may have reported the time that they get into bed, in which case actual sleep onset would be even further delayed[41].

Social media use was also associated with later wake times on school days (for both high and very high users) and on free days (for very high users). This overall pattern of later sleep timing amongst heavier social media users could be driven partly by circadian factors, if adolescents with a natural preference for later sleep timing use social media to fill time in the late evening until they feel sleepy. This possibility merits further investigation. Alternatively, this later sleep timing could suggest that heavier social media users may compensate for later sleep onset with later wake times that still allow sufficient sleep. This compensation may be possible on free days, with flexible rise times. However, on school days only 4% of adolescents reported late wake times (after 8am), as fixed rise times mean that later sleep onset effectively equates to shorter sleep opportunity on school days [6 40]. Consequently, these slightly later rise times are unlikely to fully compensate for delayed sleep onset on school days and suggest sleep restriction in a population where sleep need is

For peer review only

high [6]. Across the sample, this observed pattern of later sleep onset and rise times on free days compared to school days is consistent with well-established delays to the circadian rhythm during this developmental period[42 43], with growing pressure on policymakers to delay school start times to better align with adolescent body clocks[44].

Delayed sleep onset is therefore a key issue to target in relation to adolescents' social media use. The current cross-sectional study cannot establish causality; however, some adolescents do report delaying bedtimes as a result of social media use [13 45]. Adolescent sleep interventions should therefore consider assessing the impact of social media use on sleep schedules as standard. Further research can explore adolescents' motivations for prioritising social media over other needs, including sleep[13], and identify factors that lead some individuals to struggle with this more than others. This can inform efforts to effectively support young people to balance online interactions (and the benefits they can offer; [39 46 47]) with an appropriate and consistent sleep schedule across the week, particularly to allow sufficient sleep on school nights. By helping to combat insufficient sleep, this can have a positive impact on adolescent physical and mental health, daytime functioning and academic performance, addressing a significant health and educational burden [6].

In terms of sleep quality, very high social media users were more likely to experience nighttime awakenings than comparable average users, whereas the effect for long sleep onset latency was fully explained by covariates. Previous studies have found a significant association between social media use and measures of sleep disturbance (including long sleep onset latency and difficulty falling asleep) when controlling for: age and sex [19]; sociodemographic measures [21]; and sleep hygiene behaviours [15]. The current more extensive set of covariates also included measures of psychological wellbeing (depression and psychosocial adjustment), which were strong predictors of long sleep onset latency and have been shown to be linked to generic screentime in previous work [39]. Therefore, considering previous and current findings together, this suggests that although adolescents who spend more time on social media do tend to take longer to fall asleep, both these behaviours could reflect underlying aspects of wellbeing, with depression and anxiety linked to both poor sleep quality and social media use [16]. This is consistent with evidence that

sleep onset latency and pre-sleep cognitive arousal is predicted by underlying concerns about potentially missing out, rather than social media behaviour itself [14]. Since the purpose of this study was to isolate and quantify associations between social media use and sleep, the current approach of including wellbeing measures as covariates provided this insight into which sleep associations do and do not persist independent of wellbeing and other covariates. However, future studies can specifically examine in more detail which aspects of mental health and wellbeing may mediate or moderate these associations. Given the increasing recognition of sleep and mental health as two inextricably linked aspects of health [48], the current findings lay the foundation for more complex model testing to examine the likely bidirectional and interactive effects between social media use, sleep, mental health and other associated measures, such as school performance. Applying this approach to longitudinal and experimental data will be particularly valuable to elucidate these complex mechanisms, and to build a more holistic and balanced understanding of social media's links to both positive and negative aspects of health and wellbeing.

In contrast, the association between social media use and nighttime awakenings was only partly explained by covariates, with very high social media users still 28% more likely to have frequent difficulties with nighttime awakenings than comparable average users. Social media notification alerts may disrupt sleep during the night, particularly if users then respond by re-engaging with social media. Adolescents who use social media more also tend to have a stronger emotional connection to platforms and experience more fear of missing out [14 49]. Therefore, it is possible that very high users are more likely to remain vigilant for incoming social media alerts or to respond to these during the night, increasing arousal and contributing to difficulties falling asleep again. Further research can focus on this type of specific social media behaviours during the night, to examine whether they explain the link between higher overall use and nighttime awakenings. If incoming alerts are indeed mostly responsible, interventions can promote simple practical steps such as setting 'do not disturb' periods on social media apps.

Limitations

These findings should be considered within the limitations of the current study. Given the broad scope of the UK Millennium Cohort Study, sleep and social media use were measured

For peer review only

using individual questions rather than validated multi-item questionnaires. This limits the current analyses to a single measure of social media use - defined as the amount of time spent using social media on a typical day - which does not capture the different experiences of individual users,[50] for example in terms of content, context, timing and emotional engagement. Future research should carefully consider a range of measures to provide a more holistic view of adolescents' experiences of using social media, particularly since evidence highlights the importance of emotional and cognitive aspects of social media use for sleep [14 16]. To support future research, there is a clear need to establish *validated* measurement tools that move beyond hours per day to capture these more nuanced aspects of social media engagement. This is a key area for future development, as available tools limit the scope of potential research questions, conclusions and recommendations. Improved measurement tools moving forward can enhance understanding of the mechanisms linking social media use and sleep, as well as providing a more balanced view of both positive and negative impacts of social media experiences.

These analyses make use of six available reported sleep parameters from this representative cohort, which do allow a rounded picture of timing and quality, but future research would benefit from including validated measures of sleep duration and quality, as well as circadian preference. The current self-reported sleep measures offer valuable insight into adolescents' *subjective experience* of sleep, which is one important component of sleep, but this can diverge from verifiable objective measures of sleep parameters provided by other methods, such as the gold-standard polysomnography [51 52]. In particular, sleep state misperception in both good and poor sleepers can result in poor self-report estimates of sleep onset latency [52 53]. The current analyses therefore contribute one part of the picture, with a continued need to triangulate insight from multiple methodologies (both subjective and objective) to build a more nuanced, holistic understanding of adolescent social media use and sleep [51 54].

Furthermore, this study presents cross-sectional data, which precludes conclusions of causality. Cross-sectional analyses are prevalent in this research area, with calls for longitudinal and experimental work to enrich current understanding[55 56]. Recent studies on general technology use have supported bidirectional links between higher technology use

For peer review only

and shorter sleep in adolescents[57], with evidence that restricting phone access can advance bedtimes and extend sleep opportunity[58]. Restricting adolescent social media use is likely to be especially challenging, as this is a developmental period of increasing autonomy during which peer interactions, belonging and acceptance are highly valued [2]. Therefore, an important avenue for future research is establishing how best to support young people to balance these rewarding online social interactions with an appropriate and consistent sleep schedule, to optimise associated health- and school- outcomes.

Finally, we note that research in this area is constantly contending with rapidly evolving social media platforms and associated norms and expectations for online interactions. This can be particularly challenging for this type of large national cohort data, which in this case provides a snapshot of UK adolescents' social media use in 2015.

Conclusions

This study provides robust evidence on associations between social media use and sleep outcomes, controlling for an extensive range of covariates, in a large nationally representative sample of UK adolescents. It provides a normative profile of adolescent social media use and sleep in the UK, which can be used as a baseline to support evidence-based decision-making policy and practice rather than relying on assumptions around prevalence of high social media use. The findings indicate statistically and practically significant associations between social media use and sleep patterns, particularly late sleep onset. Future research should explore the context and experience of time using social media, to inform more meaningful discussions around best practice and updating sleep education and interventions to meet the needs of today's society. Interventions should focus on addressing delayed sleep onset, by supporting young people to balance online social interactions with an appropriate and consistent sleep schedule that allows sufficient sleep on school nights, with benefits for health and educational outcomes.

For peer review only

STATEMENTS RELATING TO THIS MANUSCRIPT

Acknowledgements

The authors would like to thank A Przybylski and J Lewsey for helpful discussions when planning methodology.

Statement of competing interests

Competing interests: All authors have completed the ICMJE uniform disclosure form at www.icmje.org/coi_disclosure.pdf and declare: no support from any organisation for the submitted work; no financial relationships with any organisations that might have an interest in the submitted work in the previous three years; no other relationships or activities that could appear to have influenced the submitted work.

Funding statement

This study was funded by an Economic and Social Research Council +3 PhD studentship for HS. The Millennium Cohort Study was funded by the Economic and Social Research Council.

Contributorship statement

HS designed the study and carried out data analysis. HS, HCW and SB interpreted the findings. HS drafted the manuscript in consultation with HCW. HCW and SB revised the manuscript for important intellectual content. All authors approve the submitted manuscript and agree to be accountable for all aspects of the work.

Ethics approval

The Millennium Cohort Study Sweep 6 was approved by the London Multicentre Research Ethics Committee (13/LO/1786).

Data availability statement

Data from the sixth sweep of the Millennium Cohort Study are available on the UK Data Service (DOI: 10.5255/UKDA-SN-8156-4).

Patient and Public Involvement

The public were not involved in designing this secondary data analysis or writing this manuscript.

For peer review only

Transparency statement

The lead author and guarantor, Holly Scott, affirms that this manuscript is an honest, accurate, and transparent account of the study being reported; that no important aspects of the study have been omitted; and that any discrepancies from the study as planned have been explained.

Dissemination declaration

This work presents secondary analysis of data deposited in the UK Data Service. It is therefore not possible for the authors to share the current findings directly with original study participants.

Exclusive licence

The Corresponding Author, Holly Scott, has the right to grant on behalf of all authors and does grant on behalf of all authors, a worldwide licence to the Publishers and its licensees in perpetuity, in all forms, formats and media (whether known now or created in the future), to i) publish, reproduce, distribute, display and store the Contribution, ii) translate the Contribution into other languages, create adaptations, reprints, include within collections and create summaries, extracts and/or, abstracts of the Contribution, iii) create any other derivative work(s) based on the Contribution, iv) to exploit all subsidiary rights in the Contribution, v) the inclusion of electronic links from the Contribution to third party material where-ever it may be located; and, vi) licence any third party to do any or all of the above.

For peer review only

REFERENCES

1. UK House of Commons. Written evidence submitted by the Royal College of Paediatrics and Child Health (SMH0156). In: Science and Technology Committee, ed. UK, 2018.
2. Blakemore S-J. Avoiding Social Risk in Adolescence. *Current Directions in Psychological Science* 2018;27(2):116-22. doi: 10.1177/0963721417738144
3. Gruber R, Wiebe ST, Wells SA, et al. Sleep and academic success: mechanisms, empirical evidence, and interventional strategies. *Adolescent medicine: state of the art reviews* 2010;21(3):522-41, x. [published Online First: 2011/02/10]
4. Perry GS, Patil SP, Presley-Cantrell LR. Raising awareness of sleep as a healthy behavior. *Prev Chronic Dis* 2013;10:E133-E33. doi: 10.5888/pcd10.130081
5. Walker M. Why we sleep: The new science of sleep and dreams. London: Penguin UK 2018.
6. Owens J. Insufficient Sleep in Adolescents and Young Adults: An Update on Causes and Consequences. *Pediatrics* 2014;134(3):E921-E32. doi: 10.1542/peds.2014-1696
7. Gradisar M, Wolfson AR, Harvey AG, et al. The sleep and technology use of Americans: findings from the National Sleep Foundation's 2011 Sleep in America poll. *Journal of clinical sleep medicine : JCSM : official publication of the American Academy of Sleep Medicine* 2013;9(12):1291-9. doi: 10.5664/jcsm.3272 [published Online First: 2013/12/18]
8. Eaton DK, McKnight-Eily LR, Lowry R, et al. Prevalence of Insufficient, Borderline, and Optimal Hours of Sleep Among High School Students – United States, 2007. *J Adolescent Health* 2010;46(4):399-401. doi: https://doi.org/10.1016/j.jadohealth.2009.10.011
9. Cain N, Gradisar M. Electronic media use and sleep in school-aged children and adolescents: A review. *Sleep Med* 2010;11(8):735-42. doi: 10.1016/j.sleep.2010.02.006
10. Vernon L, Barber BL, Modecki KL. Adolescent Problematic Social Networking and School Experiences: The Mediating Effects of Sleep Disruptions and Sleep Quality. *CyberPsychology, Behavior & Social Networking* 2015;18(7):386-92. doi: 10.1089/cyber.2015.0107
11. Przybylski AK. Digital Screen Time and Pediatric Sleep: Evidence from a Preregistered Cohort Study. *The Journal of Pediatrics* 2018 doi: 10.1016/j.jpeds.2018.09.054
12. Hale L, Guan S. Screen time and sleep among school-aged children and adolescents: a systematic literature review. *Sleep Med Rev* 2015;21:50-8. doi: 10.1016/j.smrv.2014.07.007 [published Online First: 2014/09/07]
13. Scott H, Biello S, Woods HC. Identifying drivers for bedtime social media use despite sleep costs: the adolescent perspective. . 12 Oct 2018 12 October 2018. https://doi.org/10.31234/osf.io/2xb36.
14. Scott H, Woods HC. Fear of missing out and sleep: Cognitive behavioural factors in adolescents' nighttime social media use. *Journal of Adolescence* 2018;68:61-65. doi: https://doi.org/10.1016/j.adolescence.2018.07.009
15. Arora T, Broglia E, Thomas GN, et al. Associations between specific technologies and adolescent sleep quantity, sleep quality, and parasomnias. *Sleep Med* 2014;15(2):240-47. doi: https://doi.org/10.1016/j.sleep.2013.08.799

For peer review only

16. Woods HC, Scott H. #Sleepyteens: Social media use in adolescence is associated with poor sleep quality, anxiety, depression and low self-esteem. *J Adolesc* 2016;51:41-9. doi: 10.1016/j.adolescence.2016.05.008
17. Levenson JC, Shensa A, Sidani JE, et al. Social media use before bed and sleep disturbance among young adults in the United States: a nationally representative study. *Sleep* 2017;40(9):zsx113-zsx13. doi: 10.1093/sleep/zsx113
18. Chang A-M, Aeschbach D, Duffy JF, et al. Evening use of light-emitting eReaders negatively affects sleep, circadian timing, and next-morning alertness. *Proceedings of the National Academy of Sciences* 2015;112(4):1232-37. doi: 10.1073/pnas.1418490112
19. Harbard E, Allen NB, Trinder J, et al. What's Keeping Teenagers Up? Prebedtime Behaviors and Actigraphy-Assessed Sleep Over School and Vacation. *J Adolescent Health* 2016;58(4):426-32. doi: 10.1016/j.jadohealth.2015.12.011
20. An J, Sun Y, Wan Y, et al. Associations between problematic internet use and adolescents' physical and psychological symptoms: possible role of sleep quality. *Journal of addiction medicine* 2014;8(4):282-7. doi: 10.1097/adm.0000000000000026 [published Online First: 2014/07/16]
21. Levenson JC, Shensa A, Sidani JE, et al. The association between social media use and sleep disturbance among young adults. *Prev Med* 2016;85:36-41. doi: 10.1016/j.ypmed.2016.01.001
22. Pew Research Center. Social Media Use in 2018, March 2018.
23. Centre for Longitudinal Studies. Millennium Cohort Study Sixth Sweep (MCS6): Technical Report: UCL Institute of Education, 2017.
24. Goodman R. Psychometric Properties of the Strengths and Difficulties Questionnaire. *Journal of the American Academy of Child & Adolescent Psychiatry* 2001;40(11):1337-45. doi: 10.1097/00004583-200111000-00015
25. Angold A, Costello EJ, Messer SC, et al. Development of a short questionnaire for use in epidemiological studies of depression in children and adolescents. *International Journal of Methods in Psychiatric Research* 1995;5(4):237-49.
26. Rosenberg M. Society and the adolescent self-image. 1965
27. Lichstein KL, Durrence HH, Taylor DJ, et al. Quantitative criteria for insomnia. *Behav Res Ther* 2003;41(4):427-45. doi: 10.1016/s0005-7967(02)00023-2
28. srvyr: 'dplyr'-like syntax for summary statistics of survey data. [program]. 0.3.0 version, 2018.
29. Lumley T. Analysis of Complex Survey Samples. *2004* 2004;9(8):19. doi: 10.18637/jss.v009.i08 [published Online First: 2004-01-08]
30. survey: analysis of complex survey samples [program]. 3.32 version, 2017.
31. Sterne JAC, White IR, Carlin JB, et al. Multiple imputation for missing data in epidemiological and clinical research: potential and pitfalls. *BMJ* 2009;338 doi: 10.1136/bmj.b2393
32. van Buuren S, Groothuis-Oudshoorn K. mice: Multivariate Imputation by Chained Equations in R. *J Stat Softw* 2011;45(3):1-67.
33. Zhang J, Yu KF. What's the relative risk? A method of correcting the odds ratio in cohort studies of common outcomes. *Jama* 1998;280(19):1690-1. [published Online First: 1998/12/01]
34. Pew Research Center. Teens' social media habits and experiences, November 2018.

For peer review only

35. Herring SC, Kapidzic S. Teens, Gender, and Self-Presentation in Social Media. In: Wright JD, ed. International Encyclopedia of the Social & Behavioral Sciences (Second Edition). Oxford: Elsevier 2015:146-52.
36. Galland BC, Short MA, Terrill P, et al. Establishing normal values for pediatric nighttime sleep measured by actigraphy: a systematic review and meta-analysis. *Sleep* 2018;41(4) doi: 10.1093/sleep/zsy017 [published Online First: 2018/03/29]
37. Hysing M, Pallesen S, Stormark KM, et al. Sleep patterns and insomnia among adolescents: a population-based study. *J Sleep Res* 2013;22(5):549-56. doi: 10.1111/jsr.12055 [published Online First: 2013/04/25]
38. Kuss DJ, Griffiths MD. Online Social Networking and Addiction—A Review of the Psychological Literature. *International Journal of Environmental Research and Public Health* 2011;8(9) doi: 10.3390/ijerph8093528
39. Przybylski AK, Weinstein N. A Large-Scale Test of the Goldilocks Hypothesis: Quantifying the Relations Between Digital-Screen Use and the Mental Well-Being of Adolescents. *Psychological Science* 2017;28(2):204-15. doi: 10.1177/0956797616678438
40. Asarnow LD, McGlinchey E, Harvey AG. The effects of bedtime and sleep duration on academic and emotional outcomes in a nationally representative sample of adolescents. *The Journal of adolescent health : official publication of the Society for Adolescent Medicine* 2014;54(3):350-6. doi: 10.1016/j.jadohealth.2013.09.004 [published Online First: 2013/11/15]
41. Exelmans L, Van den Bulck J. Bedtime, shuteye time and electronic media: sleep displacement is a two-step process. *J Sleep Res* 2017;26(3):364-70. doi: 10.1111/jsr.12510
42. Carskadon MA. Sleep in adolescents: the perfect storm. *Pediatric clinics of North America* 2011;58(3):637-47. doi: 10.1016/j.pcl.2011.03.003
43. Roenneberg T, Kuehnle T, Pramstaller PP, et al. A marker for the end of adolescence. *Current Biology* 2004;14(24):R1038-R39. doi: http://dx.doi.org/10.1016/j.cub.2004.11.039
44. Owens J, Drobnich D, Baylor A, et al. School Start Time Change: An In-Depth Examination of School Districts in the United States. *Mind, Brain, and Education* 2014;8(4):182-213. doi: 10.1111/mbe.12059
45. Espinoza G, Juvonen J. The Pervasiveness, Connectedness, and Intrusiveness of Social Network Site Use Among Young Adolescents. *Cyberpsych Beh Soc N* 2011;14(12):705-09. doi: 10.1089/cyber.2010.0492
46. Davis K. Friendship 2.0: adolescents' experiences of belonging and self-disclosure online. *J Adolesc* 2012;35(6):1527-36. doi: 10.1016/j.adolescence.2012.02.013
47. Davis K. Young people's digital lives: The impact of interpersonal relationships and digital media use on adolescents' sense of identity. *Computers in Human Behavior* 2013;29(6):2281-93. doi: https://doi.org/10.1016/j.chb.2013.05.022
48. Reynolds CF, 3rd, O'Hara R. DSM-5 sleep-wake disorders classification: overview for use in clinical practice. *The American journal of psychiatry* 2013;170(10):1099-101. doi: 10.1176/appi.ajp.2013.13010058 [published Online First: 2013/10/03]
49. Przybylski AK, Murayama K, DeHaan CR, et al. Motivational, emotional, and behavioral correlates of fear of missing out. *Computers in Human Behavior* 2013;29(4):1841-48. doi: 10.1016/j.chb.2013.02.014

For peer review only

50. Fox J, Moreland JJ. The dark side of social networking sites: An exploration of the relational and psychological stressors associated with Facebook use and affordances. *Computers in Human Behavior* 2015;45:168-76. doi: 10.1016/j.chb.2014.11.083
51. Tubbs AS, Dollish HK, Fernandez F, et al. Chapter 1 - The basics of sleep physiology and behavior. In: Grandner MA, ed. Sleep and Health: Academic Press 2019:3-10.
52. Fernandez-Mendoza J, Calhoun SL, Bixler EO, et al. Sleep misperception and chronic insomnia in the general population: role of objective sleep duration and psychological profiles. *Psychosomatic medicine* 2011;73(1):88-97. doi: 10.1097/PSY.0b013e3181fe365a [published Online First: 2010/10/28]
53. Silva GE, Goodwin JL, Sherrill DL, et al. Relationship between reported and measured sleep times: the sleep heart health study (SHHS). *Journal of clinical sleep medicine : JCSM : official publication of the American Academy of Sleep Medicine* 2007;3(6):622-30.
54. Gregory AM, Sadeh A. Sleep, emotional and behavioral difficulties in children and adolescents. *Sleep Med Rev* 2012;16(2):129-36. doi: https://doi.org/10.1016/j.smrv.2011.03.007
55. Bartel K, Gradisar M. New Directions in the Link Between Technology Use and Sleep in Young People. In: Nevšímalová S, Bruni O, eds. Sleep Disorders in Children. Cham: Springer International Publishing 2017:69-80.
56. Bhat S, Pinto-Zipp G, Upadhyay H, et al. "To sleep, perchance to tweet": in-bed electronic social media use and its associations with insomnia, daytime sleepiness, mood, and sleep duration in adults. *Sleep Health* 2018;4(2):166-73. doi: https://doi.org/10.1016/j.sleh.2017.12.004
57. Mazzer K, Bauducco S, Linton SJ, et al. Longitudinal associations between time spent using technology and sleep duration among adolescents. *J Adolesc* 2018;66:112-19. doi: 10.1016/j.adolescence.2018.05.004 [published Online First: 2018/05/31]
58. Bartel K, Scheeren R, Gradisar M. Altering Adolescents' Pre-Bedtime Phone Use to Achieve Better Sleep Health. *Health Communication* 2018:1-7. doi: 10.1080/10410236.2017.1422099

For peer review only

Supplementary Materials

Full breakdown of sleep patterns

Supplementary Table 1. Typical sleep onset (school days) for males & females

Typical sleep onset on school days	Male		Female		Total	
	%	n	%	n	%	n
Before 9 pm	5.8	288	4.7	248	5.2	536
9 - 9:59 pm	28.5	1619	30.0	1681	29.2	3300
10 - 10:59 pm	40.2	2352	38.8	2284	39.5	4636
11 pm - midnight	18.7	1076	20.3	1220	19.5	2296
After midnight	6.9	366	6.1	335	6.5	701

Notes: percentages account for survey design and weights.

Supplementary Table 2. Typical sleep onset (free days) for males & females

Typical sleep onset on free days	Male		Female		Total	
	%	n	%	n	%	n
Before 9 pm	0.9	53	0.9	45	0.9	98
9 - 9:59 pm	6.0	328	5.4	299	5.7	627
10 - 10:59 pm	22.8	1311	24.4	1407	23.6	2718
11 pm - midnight	35.1	2044	37.2	2208	36.1	4252
After midnight	35.2	1969	32.1	1808	33.7	3777

Notes: percentages account for survey design and weights. Gender difference in sleep onset (free days) $p < .05$.

Supplementary Table 3. Typical wake time (school days) for males & females

Typical wake time on school days	Male		Female		Total	
	%	n	%	n	%	n
Before 6 am	4.4	220	4.5	230	4.5	450
6 - 6:59 am	37.0	1995	47.9	2609	42.2	4604
7 - 7:59 am	53.3	3205	44.9	2771	49.3	5976
8 - 8:59 am	4.0	247	2.1	130	3.1	377
After 9 am	1.3	42	0.6	32	1.0	74

Notes: percentages account for survey design and weights. Gender difference in wake time (school days) $p < .001$.

For Peer review only

Supplementary Table 4. Typical wake time (free days) for males & females

Typical wake time on free days	Male		Female		Total	
	%	n	%	n	%	n
Before 8 am	9.9	545	6.4	342	8.2	887
8 - 8:59 am	17.1	940	15.6	846	16.3	1786
9 - 9:59 am	22.9	1348	26.5	1537	24.6	2885
10 - 10:59 am	27.6	1628	30.2	1763	28.8	3391
11 - 11:59 am	14.8	836	15.3	934	15.0	1770
After midday	7.8	401	6.2	348	7.0	749

Notes: percentages account for survey design and weights. Gender difference in wake time (free days) $p < .001$.

Supplementary Table 5. Typical sleep onset latency for males & females

Typical sleep onset latency	Male		Female		Total	
	%	n	%	n	%	n
0 - 15 minutes	37.1	2104	29.3	1709	33.3	3813
16 - 30 minutes	31.4	1838	33.7	1963	32.6	3801
31 - 45 minutes	14.8	819	17.3	973	16.0	1792
46 - 60 minutes	6.4	383	8.7	495	7.5	878
More than 60 minutes	10.3	516	11.0	610	10.6	1126

Notes: percentages account for survey design and weights. Gender difference in sleep onset latency $p < .001$.

Supplementary Table 6. Frequency of nighttime awakenings for males & females

Frequency of nighttime awakenings	Male		Female		Total	
	%	n	%	n	%	n
None of the time	35.8	2050	24.1	1435	30.1	3485
A little of the time	32.8	1951	32.7	1966	32.8	3917
Some of the time	14.5	831	17.6	991	16.0	1822
A good bit of the time	7.0	380	10.7	583	8.8	963
Most of the time	6.7	337	10.7	554	8.6	891
All of the time	3.1	143	4.3	226	3.7	369

Notes: percentages account for survey design and weights. Gender difference in nighttime awakenings $p < .001$.

For peer review only

Social media use by demographics

Table 1 in the main text provides a breakdown of social media use by gender. Supplementary tables 7 and 8 provide a breakdown of social media use by additional demographics: household income and ethnic minority status.

Supplementary Table 7. Social media use by household equivalised income

		Social media use				
		Low <1 h	Average 1 to <3 h	High 3 to <5 h	Very high 5+ h	**Total n**
**Household equivalized income**	Quintile 1 (lowest)	33.9	28.7	11.3	26.1	2038
	Quintile 2	30.6	29.2	16.3	23.9	2008
	Quintile 3	31.6	30.3	15.2	22.9	2414
	Quintile 4	32.4	35.8	14.0	17.9	2727
	Quintile 5 (highest)	40.0	33.8	12.7	13.6	2685

Notes: Numbers represent row percentages (e.g. 33.9% of cohort members in lowest income quintile were low social media users). Percentages account for survey design and weights. Income difference in social media use $p < .001$.

Supplementary Table 8. Social media use by ethnicity

		Social media use				
		Low <1 h	Average 1 to <3 h	High 3 to <5 h	Very high 5+ h	**Total n**
**Ethnicity**	White	32.9	31.7	14.4	21.1	9086
	Non-white	37.3	31.4	11.9	19.4	2326

Notes: Numbers represent row percentages (e.g. 32.9% of White cohort members were low social media users). Percentages account for survey design and weights. Ethnicity difference in social media use $p < .05$.

For peer review only

Survey questions

About what time do you usually **go to sleep** on a **school night**?
1 Before 9 pm
2 9 - 9:59 pm
3 10 – 10:59 pm
4 11 - midnight
5 After midnight

About what time do you usually **wake up** in the morning on a **school day**?
1 Before 6 am
2 6 - 6:59 am
3 7 – 7:59 am
4 8 - 8:59 am
5 After 9 am

About what time do you usually **go to sleep** on the nights when you **do not have school** the next day?
1 Before 9 pm
2 9 - 9:59 pm
3 10 - 10:59 pm
4 11 - midnight
5 After midnight

About what time do you **wake up** in the morning on the days when you **do not have school**?
1 Before 8 am
2 8 - 8:59 am
3 9 – 9:59 am
4 10 - 10:59 am
5 11 - 11:59 am
6 After Midday

During the last four weeks, how long did it usually take for you to fall asleep?
1 0-15 minutes
2 16-30 minutes
3 31-45 minutes
4 46-60 minutes
5 More than 60 minutes

During the last four weeks, how often did you awaken during your sleep time and have trouble falling back to sleep again?
1 All of the time
2 Most of the time
3 A good bit of the time
4 Some of the time
5 A little of the time
6 None of the time

On a normal week day during term time, how many hours do you spend on social networking or messaging sites or Apps on the internet such as Facebook, Twitter and WhatsApp?
1 None
2 Less than half an hour
3 Half an hour to less than 1 hour
4 1 hour to less than 2 hours
5 2 hours to less than 3 hours
6 3 hours to less than 5 hours

For peer review only

7 5 hours to less than 7 hours
8 7 hours or more

For peer review only

Figures

Figure 1. Odds Ratios of sleep outcomes for low, high & very high daily social media users

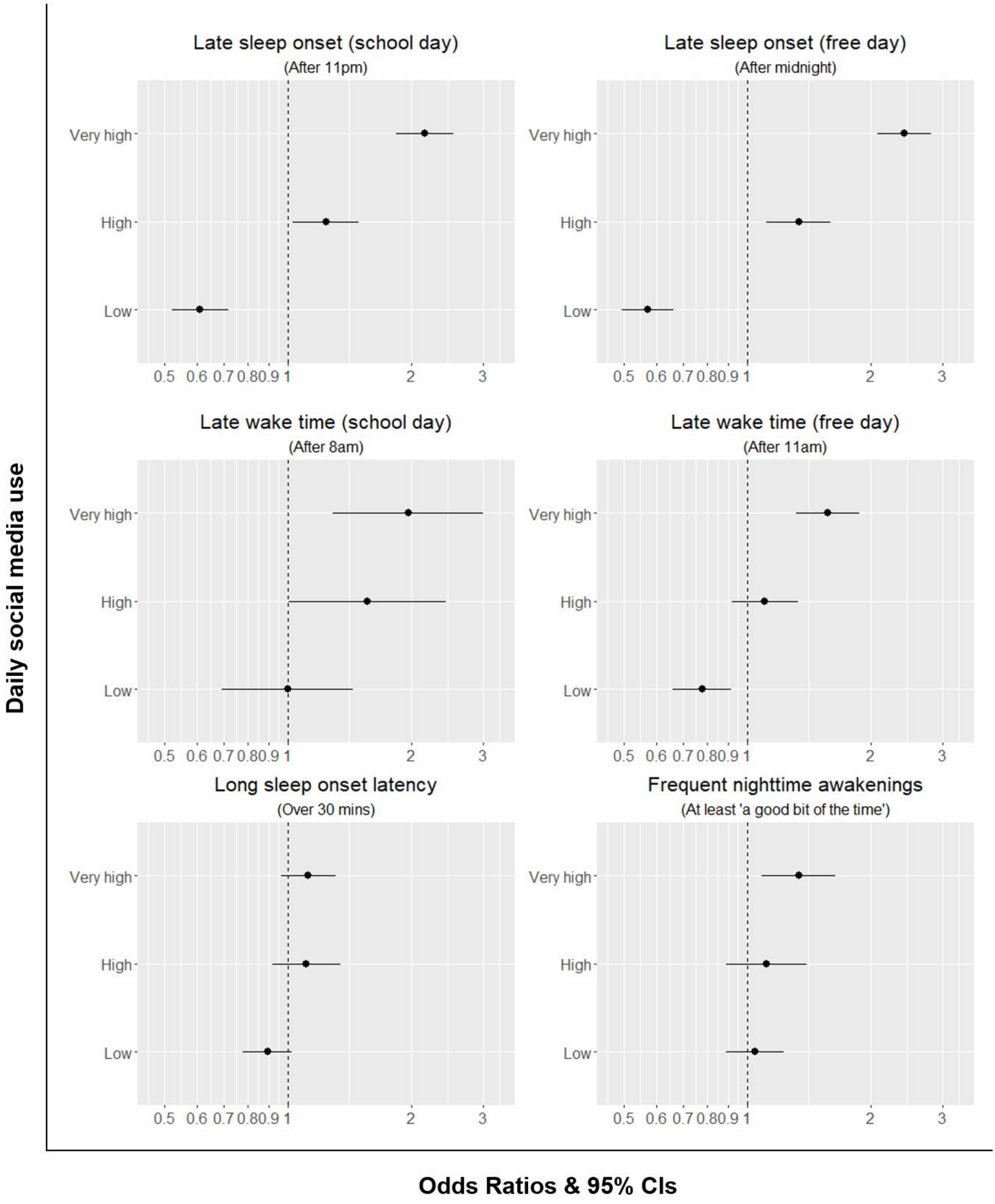

Notes: Odds Ratios measure how much higher or lower the odds of a given sleep outcome are for each category of social media user (Low, <1h; High, 3-5h; Very high, 5+h) compared to typical users (1-3h). Odds Ratios and 95% Confidence Intervals greater than 1 indicate higher odds; those below 1 indicate lower odds. Odds Ratios are adjusted to control for: exact age, sex, ethnic minority status, family income, number of siblings in household, presence of both parents, parent age, Strengths and Difficulties score, Mood and Feelings score, self-esteem, general health, social support and physical activity.

STROBE Statement—Checklist of items that should be included in reports of ***cross-sectional studies***

	Item No	Recommendation	Page No
**Title and abstract**	1	(*a*) Indicate the study's design with a commonly used term in the title or the abstract	1
		(*b*) Provide in the abstract an informative and balanced summary of what was done and what was found	2
**Introduction**			
Background/rationale	2	Explain the scientific background and rationale for the investigation being reported	4-6
Objectives	3	State specific objectives, including any prespecified hypotheses	5-6
**Methods**			
Study design	4	Present key elements of study design early in the paper	7
Setting	5	Describe the setting, locations, and relevant dates, including periods of recruitment, exposure, follow-up, and data collection	7
Participants	6	(*a*) Give the eligibility criteria, and the sources and methods of selection of participants	7
Variables	7	Clearly define all outcomes, exposures, predictors, potential confounders, and effect modifiers. Give diagnostic criteria, if applicable	7-8
Data sources/ measurement	8*	For each variable of interest, give sources of data and details of methods of assessment (measurement). Describe comparability of assessment methods if there is more than one group	7-8
Bias	9	Describe any efforts to address potential sources of bias	NA
Study size	10	Explain how the study size was arrived at	7
Quantitative variables	11	Explain how quantitative variables were handled in the analyses. If applicable, describe which groupings were chosen and why	8
Statistical methods	12	(*a*) Describe all statistical methods, including those used to control for confounding	9
		(*b*) Describe any methods used to examine subgroups and interactions	NA
		(*c*) Explain how missing data were addressed	9
		(*d*) If applicable, describe analytical methods taking account of sampling strategy	9
		(*e*) Describe any sensitivity analyses	9
**Results**			
Participants	13*	(a) Report numbers of individuals at each stage of study—eg numbers potentially eligible, examined for eligibility, confirmed eligible, included in the study, completing follow-up, and analysed	7
		(b) Give reasons for non-participation at each stage	NA
		(c) Consider use of a flow diagram	NA
Descriptive data	14*	(a) Give characteristics of study participants (eg demographic, clinical, social) and information on exposures and potential confounders	8-9
		(b) Indicate number of participants with missing data for each variable of interest	10
Outcome data	15*	Report numbers of outcome events or summary measures	8-9
Main results	16	(*a*) Give unadjusted estimates and, if applicable, confounder-adjusted estimates and their precision (eg, 95% confidence interval). Make clear which confounders were adjusted for and why they were included	10-12

For peer review only - http://bmjopen.bmj.com/site/about/guidelines.xhtml

		(*b*) Report category boundaries when continuous variables were categorized	8
		(*c*) If relevant, consider translating estimates of relative risk into absolute risk for a meaningful time period	NA
Other analyses	17	Report other analyses done—eg analyses of subgroups and interactions, and sensitivity analyses	NA
**Discussion**			
Key results	18	Summarise key results with reference to study objectives	13
Limitations	19	Discuss limitations of the study, taking into account sources of potential bias or imprecision. Discuss both direction and magnitude of any potential bias	15-16
Interpretation	20	Give a cautious overall interpretation of results considering objectives, limitations, multiplicity of analyses, results from similar studies, and other relevant evidence	14-15
Generalisability	21	Discuss the generalisability (external validity) of the study results	16
**Other information**			
Funding	22	Give the source of funding and the role of the funders for the present study and, if applicable, for the original study on which the present article is based	17

*Give information separately for exposed and unexposed groups.

Note: An Explanation and Elaboration article discusses each checklist item and gives methodological background and published examples of transparent reporting. The STROBE checklist is best used in conjunction with this article (freely available on the Web sites of PLoS Medicine at http://www.plosmedicine.org/, Annals of Internal Medicine at http://www.annals.org/, and Epidemiology at http://www.epidem.com/). Information on the STROBE Initiative is available at www.strobe-statement.org.

For peer review only

BMJ Open

Social media use and adolescent sleep patterns: cross-sectional findings from the UK Millennium Cohort Study

Journal:	*BMJ Open*
Manuscript ID	bmjopen-2019-031161.R1
Article Type:	Research
Date Submitted by the Author:	16-Jul-2019
Complete List of Authors:	Scott, Holly; University of Glasgow, School of Psychology<br>Biello, Stephany; University of Glasgow, School of Psychology<br>Woods, HC; University of Glasgow, School of Psychology
**Primary Subject Heading**:	Paediatrics
Secondary Subject Heading:	Epidemiology
Keywords:	Social media use, Sleep, Adolescents, Delayed bedtime, Sleep quality, Screen time

SCHOLARONE™
Manuscripts

TITLE PAGE

Title

Social media use and adolescent sleep patterns: cross-sectional findings from the UK Millennium Cohort Study

Author names

Holly Scott, MSc; Stephany M Biello, PhD; Heather Cleland Woods, PhD

Author job titles & affiliation address

Holly Scott, PhD candidate

Stephany M Biello, Professor & Depute Head of School

Heather Cleland Woods, Lecturer

All authors have same institutional affiliation/address:

School of Psychology, University of Glasgow, 62 Hillhead Street, Glasgow, G12 8QB.

Corresponding author

Holly Scott, h.scott.1@research.gla.ac.uk, postal address as above.

For peer review only

ABSTRACT

Objectives

This study examines associations between social media use and multiple sleep parameters in a large representative adolescent sample, controlling for a wide range of covariates.

Design

The authors used cross-sectional data from the Millennium Cohort Study, a large nationally representative UK birth cohort study.

Participants

Data from 11,872 adolescents (aged 13-15 years) were used in analyses.

Methods

Six self-reported sleep parameters captured sleep timing and quality: sleep onset and wake times (on school days and free days), sleep onset latency (time taken to fall asleep) and trouble falling back asleep after nighttime awakening. Binomial logistic regressions investigated associations between daily social media use and each sleep parameter, controlling for a range of relevant covariates.

Results

Average social media use was 1 to <3h per day (31.6%, n = 3720). 33.7% were classed as low users (<1h; n = 3986), 13.9% were high users (3 to <5h; n = 1602) and 20.8% were very high users (5+h; n = 2203). Girls reported spending more time on social media than boys. Overall, heavier social media use was associated with poorer sleep patterns, controlling for covariates. For example, very high social media users were more likely than comparable average users to report late sleep onset (OR = 2.14, 95% CI: 1.83 to 2.50) and wake times (OR = 1.97, 95% CI: 1.32 to 2.93) on school days, and trouble falling back asleep after nighttime awakening (OR = 1.36, 95% CI: 1.10 to 1.66).

Conclusions

This study provides a normative profile of UK adolescent social media use and sleep. Results indicate statistically and practically significant associations between social media use and sleep patterns, particularly late sleep onset. Sleep education and interventions can focus on supporting young people to balance online interactions with an appropriate sleep schedule that allows sufficient sleep on school nights..

For peer review only

STRENGTHS AND LIMITATIONS

- Provides a current normative profile of social media use and sleep in UK adolescents
- Moves beyond generic “screen time” to examine social media specifically
- Uses data from a large representative sample, including comprehensive covariates
- Uses self-reported measures of social media use and sleep patterns
- Measures only duration of social media use, rather than content and context

For peer review only

INTRODUCTION

There is significant current attention towards the possible impact of screentime and social media on our adolescents' health. However, the lack of empirical evidence to support policy and practice development in this area has been consistently voiced by clinicians and researchers. For example, at the UK House of Commons Science and Technology Committee inquiry into the impact of social media and screen-use on young people's health use in adolescence, the Royal College of Paediatrics and Child Health (RCPCH) urged the UK government as a matter of priority to develop guidance for health practitioners along the same lines as the American Academy of Paediatrics (AAP) but importantly based on UK data[1 2]. They also argue along with other researchers that there is a need to refocus away from correlations between generic terms such as "screentime" and poor wellbeing, towards meaningfully quantifying how various types of technology use impact on different areas of child and adolescent health and wellbeing. This study presents UK data that provide a nationally representative profile of current adolescent social media use, and takes a data-driven approach to quantify sleep patterns for high and very high users relative to average users.

This study focuses on sleep, which – despite often being overlooked in public health messages and education interventions[3 4] – is increasingly recognised as a key component of wider health and wellbeing[5]. Adolescent sleep is an important public health issue, as insufficient sleep is highly prevalent in this age group and has implications for mental health, obesity, academic performance and safety[6]. With the majority of adolescents reporting insufficient sleep to function properly or to meet recommended guidelines[7 8], there is growing concern that social media may be a contributing factor for today's teenagers. For example, the potential for 24/7 social media interactions may exacerbate the existing conflict of early school start times with naturally delayed adolescent rhythms and other social and educational demands[6 9 10]. As a highly relevant issue for paediatric practice, there is a clear need for UK evidence to inform and update decision making in medical practice and policy to address this current issue in adolescent sleep.

This study responds to this need, presenting large-scale UK data on adolescent sleep and social media use, whilst addressing a number of existing gaps in available international

For peer review only

evidence. In addition to providing much-needed UK evidence, the current approach also addresses the need for evidence that: (1) examines social media specifically, rather than generic screentime; (2) isolates effects for a range of sleep parameters by accounting for an extensive range of covariates; (3) frames these effects within the context of current adolescent social media norms to provide meaningful comparisons. The current approach to ensure that this evidence can meaningfully inform policy and practice by addressing each of these needs is discussed further below.

Firstly, it is important for available evidence to examine social media individually, rather than aggregating these interactions and other media use under the umbrella term "screentime". A recent large-scale US study indicated a significant but modest effect for overall screentime and sleep, and called for future research to examine effects for specific technologies [11]. In particular, the interactive nature of social media presents uniquely relevant issues for adolescent sleep compared to other forms of screentime or traditional media [7 12]. Although facilitated by screens, social media interactions are underpinned by the same drivers as any social interaction, with a desire for inclusion and belonging mixing with concerns over violating social expectations or etiquette [13]. These concerns can make it difficult to disengage from social media at bedtime, with some adolescents identifying this as a cause of delayed sleep onset and daytime tiredness [13]. These unique social and emotional aspects of online interactions underline the importance of examining social media use specifically, in relation to adolescent sleep outcomes.

Secondly, to meaningfully inform an evidence-based response to social media use, research must examine multiple sleep parameters and isolate these effects by controlling for an extensive range of relevant covariates. This is crucial to assess the practical significance of underlying direct effects [11] and to identify which aspects of adolescent sleep merit attention to social media use in practice and policy. Available research on social media use and sleep is often left questioning whether reported effects could be explained by other individual factors: for example if more anxious, depressed or sedentary adolescents may tend to both use social media more and report poorer sleep [12 14]. Individual studies that have controlled for specific groups of covariates generally suggest that associations do persist [15 16]. However, there remains a need for large-scale evidence that addresses a wide range of covariates

For peer review only

simultaneously, to more robustly establish which dimensions of sleep have a direct association with social media use and which reflect another underlying issue (e.g. anxious or depressive symptoms).

This type of evidence is required to invest time and resources effectively, by identifying which sleep complaints may benefit from directly addressing social media use. For example, a range of sleep complaints from insufficient sleep to problems initiating or maintaining sleep have been examined in relation to social media use. In terms of sleep duration, time spent using social media may displace sleep directly or displace other daytime activities (such as homework) that are then delayed and disrupt nighttime routines [9 17]. Social media use may also impact on the quality of sleep via increased arousal, not simply though light exposure [18], but particularly via cognitive and social activity [12 14 19]. Given these different potential mechanisms, it is therefore important to examine social media use in relation to a range of sleep parameters, to identify which of these links have the most practical significance after accounting for relevant factors.

Thirdly, evidence should frame these effects within the context of current norms for adolescent social media use. Research to date has tended to focus on problematic or "addicted" social media users [10 20], or to compare outcomes for the highest users against the lowest users [15 17 21]. In contrast, first establishing what constitutes typical use and then comparing outcomes for relatively higher or lower users against this reference point can support more meaningful conclusions. This data-driven approach avoids imposing arbitrary or quickly outdated cut-offs, taking into account recent rapid increases in social media use [22]. Comparing sleep patterns for higher users against average users can better support practical and realistic discussions on best practice, that consider the context of current adolescent social media norms.

This study targets these existing gaps in available international evidence, while providing much-needed large-scale UK evidence. It examines associations between social media use and multiple sleep parameters in a large, nationally representative adolescent sample: the UK Millennium Cohort Study [23]. It first investigates current norms in adolescent social media use to establish the average level of daily use and the prevalence of comparatively high use.

For peer review only

It then examines which sleep parameters are associated with social media use, isolating these effects by controlling for extensive covariates and quantifying effects for higher users relative to average users. This aims to provide rigorous and meaningful evidence to inform practice and policy to support healthy adolescent sleep and social media use.

METHODS

Participants

The UK Millennium Cohort Study is a nationally representative, multidisciplinary survey which aims to explore the influence of family context on child and adolescent development and outcomes. The survey covers a broad range of domains, such as parenting, housing, poverty and health. It consists of a random 2-stage sample drawn from all live UK births in the 12-month period starting 1 September 2000 in England and Wales and 1 December 2000 in Scotland and Northern Ireland, identified through the Child Benefit register [23]. The clustered sample is drawn from a disproportionately stratified sample of electoral wards (local areas) to provide adequate representation of areas with higher concentrations of minority ethnic and disadvantaged families. Parents completed the first survey sweep in 2001 when their child was aged 9 months, with 18,818 cohort members. Children also completed surveys from age 7 (sweep 4) onwards. The most recent survey (sweep 6 at around age 14) gathered self-report data from 11,872 cohort members, including questions on their typical social media use and sleep patterns. Parents were required to give written consent to complete the parent survey and for the interviewer to invite the cohort member to participate in the young person survey. Cohort members then also had to give verbal consent to complete the young person survey, which was self-completed on the interviewer's tablet.

Materials

The current analyses make use of available data from the UK Millennium Cohort Study, which measured social media use and sleep using single-item self-report questions. Although not validated questionnaire measures, these survey questions do provide a snapshot of the subjective experience of sleep and social media use in this large representative sample, capturing a range of sleep habits and the typical time spent using social media each day.

For peer review only

Social media use

Participants indicated how much time they spent using social media on a typical weekday, choosing from 8 response categories (ranging from 0 hours to 7+ hours) to answer the following question: "On a normal week day during term time, how many hours do you spend on social networking or messaging sites or Apps on the internet such as Facebook, Twitter and WhatsApp?"

Sleep parameters

Participants reported typical sleep habits through six single items (each with 5 or 6 response categories) that assessed: sleep onset and wake times (on school days and free days, separately), sleep onset latency (time taken to fall asleep) and trouble falling back asleep after nighttime awakening. The Supplementary Materials provide a full list of items and response categories.

Covariates

In addition, the following relevant covariates (identified based on literature) had available data in the UK Millennium Cohort Study: demographics (ethnic minority status, OECD equivalised weekly family income); family composition (number of siblings in household, presence of both parents, age of primary parent/carer responder); psychosocial adjustment (using the parent-report Strengths and Difficulties Questionnaire)[24]; depressive symptoms (using the Short Mood and Feelings Questionnaire)[25]; self-esteem (using a shortened and adapted version of the Rosenberg Self-Esteem Scale)[26]; general health (single item), social support (three items) and physical activity (single item).

Data analysis

Since the aim of the study was to compare sleep outcomes for high and low users versus average users, based on the distribution we initially collapsed responses into three categories: under 1 hour for 'low' users (33.7%), 1 to <3 hours for 'average' users (31.6%), and 3 hours or more for 'high' users (34.7%). Given the broad range covered by this 'high' user category (including responses of 3 to <5, 5 to <7 and 7+ hours), and with sufficient numbers, we separated this into 'high' (3 to <5 hours; 13.9%) and 'very high' users (5+ hours; 20.8%) to allow more detailed exploration.

For peer review only

We collapsed responses for each sleep measure into binary outcomes. For poor sleep quality, these outcomes were: sleep onset latency over 30 minutes (commonly used to indicate poor sleep quality) [27 28] and difficulty falling asleep following nighttime awakenings at least 'a good bit of the time'. For late sleep onset and wake times, we took a data-driven approach to identify meaningful cut-off points, which were defined as later than average (including responses in categories later than the median response category). Table 1 summarises the resulting criteria for each sleep outcome and associated prevalence rates.

Table 1 Social media use and sleep outcomes: criteria and prevalence

Variable	Criteria	Prevalence (%)		
		Male	Female	Total
Daily social media use				
Low	< 1 hour	43.8	22.8	33.7
Average	1 to <3 hours	32.1	31.1	31.6
High	3 to <5 hours	10.4	17.7	13.9
Very high	5+ hours	13.7	28.4	20.8
Sleep outcomes				
Late sleep onset (school day)	After 11pm	25.5	26.5	26.0
Late sleep onset (free day)	After midnight	35.2	32.1	33.7
Late wake time (school day)	After 8am	5.3	2.7	4.0
Late wake time (free day)	After 11am	22.5	21.5	22.0
Long sleep onset latency	Over 30 minutes	31.5	37.0	34.1
Trouble falling back asleep after nighttime awakening	At least 'a good bit of the time'	16.9	25.7	21.1

Notes: Percentages account for survey design and weights. Criteria for late sleep onset and wake times defined as later than the median response category. Gender difference in daily social media use, late wake time (school day), long sleep onset latency and trouble falling back asleep after nighttime awakening $p < .001$; gender difference in late sleep onset (free day) $p < .01$. For a breakdown of social media use by other demographics (household income and ethnicity), see the Supplementary Materials.

Separate binomial logistic regression models predicted Odds Ratios of each sleep outcome for low, high and very high social media users, compared to average users. We ran models that controlled only for exact age and sex, followed by models that further controlled for measures of demographics, family characteristics, psychological wellbeing and health (see Materials section for full details). All analyses allowed for the complex survey design (with

For peer review only

its clustered, stratified sample) and used longitudinal weights to account for non-random longitudinal attrition from the sample, using the 'survey' and 'srvyr' packages in R [29-31].

Multiple imputation was performed to account for missing data, reducing bias and increasing power [32]. The overall missing data rate was 2.8%, ranging from 0.0% to 6.0% for individual measures, with most measures below 5%. We make the assumption that data are missing at random (i.e. that patterns of missingness can be explained by other variables available in the data; [32]). All variables, including covariates, were used in the imputation model, which was run using R package 'mice' [33]. Estimates were combined across 10 imputed data sets (each produced through 10 iterations). Results were similar for analyses on multiply imputed and complete case data, so only multiply imputed analysis is presented here.

RESULTS

The median time spent using social media on a typical day was 1 to <3 hours (32% of adolescents); however, 21% used social media for at least 5 hours. Girls tended to use social media more than boys (see Table 1).

Median sleep onset times were 10-11pm on school days (with 26% falling asleep later than this) and between 11pm and midnight on free days (with 34% falling asleep later; see Table 1). Median wake times were 7-8am on school days (with only 4% waking later than this) and 10-11am on free days (with 22% waking later). Boys were more likely to fall asleep late on free days and wake up late on school days. In measures of poor sleep quality, 34% typically took longer than 30 minutes to fall asleep and 21% reported difficulties falling asleep following nighttime awakenings at least 'a good bit of the time'. Girls were more likely to have long sleep onset latency and trouble falling back asleep after nighttime awakening.

Separate binomial logistic regression models explored whether odds of each sleep outcome differed for low, high and very high social media users, compared to average users (1 to <3 h). First, models controlled for exact age and sex (see Table 2). Very high social media use (5+ h) was associated with higher odds of all six sleep outcomes. High social media use (3 to <5 h) was associated with higher odds of all outcomes except for late rise times on free

For peer review only

days. Low social media use (<1 h) was associated with lower odds of late sleep onset on school- and free- days, and late wake times on free days.

Table 2 Binomial logistic regressions (adjusting only for age & sex)

	**Low: <1 h**		**High: 3 to <5 h**		**Very high: 5+ h**	
	OR (95% CI)	*p*	OR (95% CI)	*p*	OR (95% CI)	*p*
Late sleep onset (school days)	0.63 (0.53, 0.75)	< .001	1.38 (1.16, 1.65)	< .001	2.75 (2.38, 3.18)	< .001
Late sleep onset (free days)	0.6 (0.51, 0.7)	< .001	1.44 (1.19, 1.74)	< .001	3.05 (2.63, 3.54)	< .001
Late wake time (school days)	1.12 (0.77, 1.62)	*ns*	1.63 (1.05, 2.53)	< .05	2.49 (1.62, 3.83)	< .001
Late wake time (free days)	0.81 (0.69, 0.96)	< .05	1.16 (0.97, 1.39)	*ns*	1.82 (1.54, 2.16)	< .001
Sleep Onset Latency > 30 mins	0.92 (0.8, 1.05)	*ns*	1.24 (1.04, 1.49)	< .05	1.48 (1.27, 1.71)	< .001
Frequent nighttime awakenings	1.06 (0.9, 1.26)	*ns*	1.31 (1.07, 1.61)	< .05	2.11 (1.75, 2.55)	< .001

Notes: See Table 1 for criteria and prevalence of each sleep outcome. Odds Ratios measure how much higher or lower the odds of a given sleep outcome are for each category of social media user (Low, <1h; High, 3 to <5h; Very high, 5+h) compared to the reference category (Average users, 1 to <3h). Odds Ratios and 95% Confidence Intervals greater than 1 indicate higher odds; those below 1 indicate lower odds. Odds Ratios are adjusted to control for exact age & sex. *ns* = non-significant.

Further modelling then controlled for a more comprehensive set of covariates (see Table 3, with note detailing list of covariates). High social media use was no longer significantly associated with long sleep onset latency or frequent nighttime awakenings. Very high social media was no longer significantly associated with long sleep onset latency; however, its association with frequent nighttime awakenings remained significant but smaller. Patterns of significant associations for late sleep onset and wake times remained unchanged, although effect sizes were reduced, particularly for very high social media use.

For peer review only

Table 3 Binomial logistic regressions (with further adjustments for covariates)

	**Low: <1 h**		**High: 3 to <5 h**		**Very high: 5+ h**	
	OR (95% CI)	*p*	OR (95% CI)	*p*	OR (95% CI)	*p*
Late sleep onset (school days)	0.61 (0.51, 0.73)	< .001	1.23 (1.02, 1.49)	< .05	2.14 (1.83, 2.5)	< .001
Late sleep onset (free days)	0.57 (0.49, 0.68)	< .001	1.32 (1.09, 1.6)	< .01	2.41 (2.08, 2.79)	< .001
Late wake time (school days)	1.04 (0.71, 1.5)	*ns*	1.56 (1.02, 2.4)	< .05	1.97 (1.32, 2.93)	< .01
Late wake time (free days)	0.79 (0.67, 0.93)	< .01	1.12 (0.92, 1.35)	*ns*	1.57 (1.32, 1.87)	< .001
Sleep Onset Latency > 30 mins	0.9 (0.78, 1.04)	*ns*	1.11 (0.92, 1.34)	*ns*	1.12 (0.96, 1.32)	*ns*
Frequent nighttime awakenings	1.04 (0.88, 1.24)	*ns*	1.08 (0.87, 1.35)	*ns*	1.36 (1.1, 1.66)	< .01

Notes: See Table 1 for criteria and prevalence of each sleep outcome. Reference category is Average (1 to <3h). Odds Ratios are adjusted to control for: exact age, sex, ethnic minority status, family income, number of siblings in household, presence of both parents in household, parent age, Strengths and Difficulties score, Mood and Feelings score, self-esteem, general health, social support and physical activity. *ns* = non-significant.

For ease of interpretation, we also transformed the resulting adjusted Odds Ratios from these covariate models into adjusted Relative Risks [34] (see Table 4). These summarise differences in *probabilities,* as opposed to odds, and can be interpreted more intuitively. For example, the adjusted relative risk of 1.68 indicates that an adolescent with very high social media use is 68% more likely to fall asleep after 11pm on school nights than a comparable adolescent (controlling for covariates) with average social media use.

Table 4 Relative Risks (from covariate-adjusted models)

	**Low: <1 h**	**High: 3 to <5 h**	**Very high: 5+ h**

For peer review only

	RR (95% CI)	*p*	RR (95% CI)	*p*	RR (95% CI)	*p*
Late sleep onset (school days)	0.67 (0.58, 0.78)	<.001	1.17 (1.02, 1.33)	<.05	1.68 (1.52, 1.84)	<.001
Late sleep onset (free days)	0.66 (0.58, 0.75)	<.001	1.2 (1.06, 1.35)	<.01	1.69 (1.57, 1.81)	<.001
Late wake time (school days)	1.03 (0.72, 1.48)	*ns*	1.54 (1.02, 2.29)	<.05	1.91 (1.3, 2.76)	<.01
Late wake time (free days)	0.83 (0.72, 0.95)	<.01	1.09 (0.94, 1.26)	*ns*	1.41 (1.24, 1.59)	<.001
Sleep Onset Latency > 30 mins	0.93 (0.84, 1.03)	*ns*	1.07 (0.94, 1.21)	*ns*	1.08 (0.97, 1.2)	*ns*
Frequent nighttime awakenings	1.03 (0.89, 1.19)	*ns*	1.07 (0.89, 1.28)	*ns*	1.28 (1.09, 1.5)	<.01

Notes: Relative Risks transformed from Odds Ratios in covariate-controlled binomial logistic regression models (see Table 3). Reference category is Average (1 to <3h). E.g. RR of 1.68 means very high users are 68% more likely to fall asleep late on school days than comparable average users. *ns* = non-significant.

DISCUSSION

This study aimed to address calls from those working in policy and practice to establish a UK data driven profile of current adolescent daily social media use, and to examine links to a key component of wider adolescent health and wellbeing using multiple sleep parameters whilst accounting for a wide range of covariates, using data from a large nationally representative sample of UK adolescents. The results highlighted a wide range of reported daily social media use, with tertiles defining low, average and high use on a typical school day as <1 hour, 1 to <3 hours and 3+ hours respectively. This indicates generally heavier social media use compared to young adults [21] and provides a current normative profile for UK adolescents. One in five adolescents were classed as very high users, spending 5+ hours using social media on a typical school day, whereas two thirds of the sample used social media for less than 3 hours. This provides a data-driven profile of use to support decision-making, rather than relying on assumptions around prevalence of high use. In line with previous studies, girls tended to spend more time on social media than boys[35 36] and report

For peer review only

poorer sleep quality [37 38]. This reinforces the importance of controlling for gender when examining these associations, and highlights the need for continued work to explore the sleep implications of *how* adolescent boys and girls spend their time on social media (with previous evidence of gender differences in preferred platforms, motivations and self-presentation [35 36 39]).

In terms of sleep timing, social media use remained significantly associated with late sleep onset and wake times after controlling for covariates, with the strongest effect for sleep onset. Very high social media users were roughly 70% more likely than comparable average users to fall asleep later than average, i.e. after 11pm on school days and after midnight on free days. Low social media users were least likely to fall asleep late, indicating that unlike mental wellbeing, optimal outcomes for sleep are associated with minimal – not moderate – use [40]. These findings are consistent with the idea that social media displaces sleep: either directly or indirectly [9 17]. Direct sleep displacement may be particularly likely on school days, especially for very high users, since limited social media access during school hours means that at least part of this daily time on social media is likely to take place close to bedtime. Bedtime social media use can delay sleep onset [14], with some adolescents reporting difficulties disengaging from social media to sleep [13]. A similar process could also indirectly delay sleep onset, if other daytime activities (e.g. homework) are delayed due to a sense of urgency to check and respond to social media notifications. This link to later sleep onset is a particular concern on school days, as late school day bedtimes longitudinally predict poorer academic and emotional outcomes [41]. Whilst the survey question aimed to measure sleep onset time by asking what time participants "go to sleep", some participants may have reported the time that they get into bed, in which case actual sleep onset would be even further delayed[42].

Social media use was also associated with later wake times on school days (for both high and very high users) and on free days (for very high users). This overall pattern of later sleep timing amongst heavier social media users could be driven partly by circadian factors, if adolescents with a natural preference for later sleep timing use social media to fill time in the late evening until they feel sleepy. This possibility merits further investigation. Alternatively, this later sleep timing could suggest that heavier social media users may

For peer review only

compensate for later sleep onset with later wake times that still allow sufficient sleep. This compensation may be possible on free days, with flexible rise times. However, on school days only 4% of adolescents reported late wake times (after 8am), as fixed rise times mean that later sleep onset effectively equates to shorter sleep opportunity on school days [6 41]. Consequently, these slightly later rise times are unlikely to fully compensate for delayed sleep onset on school days and suggest sleep restriction in a population where sleep need is high [6]. Across the sample, this observed pattern of later sleep onset and rise times on free days compared to school days is consistent with well-established delays to the circadian rhythm during this developmental period[43 44], with growing pressure on policymakers to delay school start times to better align with adolescent body clocks[45].

Delayed sleep onset is therefore a key issue to target in relation to adolescents' social media use. The current cross-sectional study cannot establish causality; however, some adolescents do report delaying bedtimes as a result of social media use [13 46]. Adolescent sleep interventions should therefore consider assessing the impact of social media use on sleep schedules as standard. Further research can explore adolescents' motivations for prioritising social media over other needs, including sleep[13], and identify factors that lead some individuals to struggle with this more than others. This can inform efforts to effectively support young people to balance online interactions (and the benefits they can offer; [40 47 48]) with an appropriate and consistent sleep schedule across the week, particularly to allow sufficient sleep on school nights. By helping to combat insufficient sleep, this can have a positive impact on adolescent physical and mental health, daytime functioning and academic performance, addressing a significant health and educational burden [6].

In terms of sleep quality, very high social media users were more likely to experience nighttime awakenings than comparable average users, whereas the effect for long sleep onset latency was fully explained by covariates. Previous studies have found a significant association between social media use and measures of sleep disturbance (including long sleep onset latency and difficulty falling asleep) when controlling for: age and sex [19]; sociodemographic measures [21]; and sleep hygiene behaviours [15]. The current more extensive set of covariates also included measures of psychological wellbeing (depression

For peer review only

and psychosocial adjustment), which were strong predictors of long sleep onset latency and have been shown to be linked to generic screentime in previous work [40]. Therefore, considering previous and current findings together, this suggests that although adolescents who spend more time on social media do tend to take longer to fall asleep, both these behaviours could reflect underlying aspects of wellbeing, with depression and anxiety linked to both poor sleep quality and social media use [16]. This is consistent with evidence that sleep onset latency and pre-sleep cognitive arousal is predicted by underlying concerns about potentially missing out, rather than social media behaviour itself [14]. Since the purpose of this study was to isolate and quantify associations between social media use and sleep, the current approach of including wellbeing measures as covariates provided this insight into which sleep associations do and do not persist independent of wellbeing and other covariates. However, future studies can specifically examine in more detail which aspects of mental health and wellbeing may mediate or moderate these associations. Given the increasing recognition of sleep and mental health as two inextricably linked aspects of health [49], the current findings lay the foundation for more complex model testing to examine the likely bidirectional and interactive effects between social media use, sleep, mental health and other associated measures, such as school performance. Applying this approach to longitudinal and experimental data will be particularly valuable to elucidate these complex mechanisms, and to build a more holistic and balanced understanding of social media's links to both positive and negative aspects of health and wellbeing.

In contrast, the association between social media use and nighttime awakenings was only partly explained by covariates, with very high social media users still 28% more likely to have frequent difficulties with nighttime awakenings than comparable average users. Social media notification alerts may disrupt sleep during the night, particularly if users then respond by re-engaging with social media. Adolescents who use social media more also tend to have a stronger emotional connection to platforms and experience more fear of missing out [14 50]. Therefore, it is possible that very high users are more likely to remain vigilant for incoming social media alerts or to respond to these during the night, increasing arousal and contributing to difficulties falling asleep again. Further research can focus on this type of specific social media behaviours during the night, to examine whether they explain the link between higher overall use and nighttime awakenings. If incoming alerts are indeed mostly

For peer review only

responsible, interventions can promote simple practical steps such as setting 'do not disturb' periods on social media apps.

Limitations

These findings should be considered within the limitations of the current study. Given the broad scope of the UK Millennium Cohort Study, sleep and social media use were measured using individual questions rather than validated multi-item questionnaires. This limits the current analyses to a single measure of social media use - defined as the amount of time spent using social media on a typical day - which does not capture the different experiences of individual users,[51] for example in terms of content, context, timing and emotional engagement. Future research should carefully consider a range of measures to provide a more holistic view of adolescents' experiences of using social media, particularly since evidence highlights the importance of emotional and cognitive aspects of social media use for sleep [14 16]. To support future research, there is a clear need to establish *validated* measurement tools that move beyond hours per day to capture these more nuanced aspects of social media engagement. This is a key area for future development, as available tools limit the scope of potential research questions, conclusions and recommendations. Improved measurement tools moving forward can enhance understanding of the mechanisms linking social media use and sleep, as well as providing a more balanced view of both positive and negative impacts of social media experiences.

These analyses make use of six available reported sleep parameters from this representative cohort, which do allow a rounded picture of timing and quality, but future research would benefit from including validated measures of sleep duration and quality, as well as circadian preference. The current self-reported sleep measures offer valuable insight into adolescents' *subjective experience* of sleep, which is one important component of sleep, but this can diverge from verifiable objective measures of sleep parameters provided by other methods, such as the gold-standard polysomnography [52 53]. In particular, sleep state misperception in both good and poor sleepers can result in poor self-report estimates of sleep onset latency, [53] although these differences in sleep onset latency estimates from self-report and polysomnography tend to be small[54]. The current analyses therefore contribute one part of the picture, with a continued need to triangulate insight from multiple

methodologies (both subjective and objective) to build a more nuanced, holistic understanding of adolescent social media use and sleep [52 55].

Furthermore, this study presents cross-sectional data, which precludes conclusions of causality. Cross-sectional analyses are prevalent in this research area, with calls for longitudinal and experimental work to enrich current understanding[56 57]. Recent studies on general technology use have supported bidirectional links between higher technology use and shorter sleep in adolescents[58], with evidence that restricting phone access can advance bedtimes and extend sleep opportunity[59]. Restricting adolescent social media use is likely to be especially challenging, as this is a developmental period of increasing autonomy during which peer interactions, belonging and acceptance are highly valued [2]. Therefore, an important avenue for future research is establishing how best to support young people to balance these rewarding online social interactions with an appropriate and consistent sleep schedule, to optimise associated health- and school- outcomes.

Finally, we note that research in this area is constantly contending with rapidly evolving social media platforms and associated norms and expectations for online interactions. This can be particularly challenging for this type of large national cohort data, which in this case provides a snapshot of UK adolescents' social media use in 2015.

Conclusions

This study provides robust evidence on associations between social media use and sleep outcomes, controlling for an extensive range of covariates, in a large nationally representative sample of UK adolescents. It provides a normative profile of adolescent social media use and sleep in the UK, which can be used as a baseline to support evidence-based decision-making policy and practice rather than relying on assumptions around prevalence of high social media use. The findings indicate statistically and practically significant associations between social media use and sleep patterns, particularly late sleep onset. Future research should explore the context and experience of time using social media, to inform more meaningful discussions around best practice and updating sleep education and interventions to meet the needs of today's society. Interventions should focus on addressing delayed sleep onset, by supporting young people to balance online

For peer review only

social interactions with an appropriate and consistent sleep schedule that allows sufficient sleep on school nights, with benefits for health and educational outcomes.

For peer review only

STATEMENTS RELATING TO THIS MANUSCRIPT

Acknowledgements

The authors would like to thank A Przybylski and J Lewsey for helpful discussions when planning methodology.

Statement of competing interests

Competing interests: All authors have completed the ICMJE uniform disclosure form at www.icmje.org/coi_disclosure.pdf and declare: no support from any organisation for the submitted work; no financial relationships with any organisations that might have an interest in the submitted work in the previous three years; no other relationships or activities that could appear to have influenced the submitted work.

Funding statement

This study was funded by an Economic and Social Research Council +3 PhD studentship for HS. The Millennium Cohort Study was funded by the Economic and Social Research Council.

Contributorship statement

HS designed the study and carried out data analysis. HS, HCW and SB interpreted the findings. HS drafted the manuscript in consultation with HCW. HCW and SB revised the manuscript for important intellectual content. All authors approve the submitted manuscript and agree to be accountable for all aspects of the work.

Ethics approval

The Millennium Cohort Study Sweep 6 was approved by the London Multicentre Research Ethics Committee (13/LO/1786).

Data availability statement

Data from the sixth sweep of the Millennium Cohort Study are available on the UK Data Service (DOI: 10.5255/UKDA-SN-8156-4).

Patient and Public Involvement

The public were not involved in designing this secondary data analysis or writing this manuscript.

For peer review only

Transparency statement

The lead author and guarantor, Holly Scott, affirms that this manuscript is an honest, accurate, and transparent account of the study being reported; that no important aspects of the study have been omitted; and that any discrepancies from the study as planned have been explained.

Dissemination declaration

This work presents secondary analysis of data deposited in the UK Data Service. It is therefore not possible for the authors to share the current findings directly with original study participants.

Exclusive licence

The Corresponding Author, Holly Scott, has the right to grant on behalf of all authors and does grant on behalf of all authors, a worldwide licence to the Publishers and its licensees in perpetuity, in all forms, formats and media (whether known now or created in the future), to i) publish, reproduce, distribute, display and store the Contribution, ii) translate the Contribution into other languages, create adaptations, reprints, include within collections and create summaries, extracts and/or, abstracts of the Contribution, iii) create any other derivative work(s) based on the Contribution, iv) to exploit all subsidiary rights in the Contribution, v) the inclusion of electronic links from the Contribution to third party material where-ever it may be located; and, vi) licence any third party to do any or all of the above.

For peer review only

REFERENCES

1. UK House of Commons. Written evidence submitted by the Royal College of Paediatrics and Child Health (SMH0156). In: Science and Technology Committee, ed. UK, 2018.
2. Blakemore S-J. Avoiding Social Risk in Adolescence. *Current Directions in Psychological Science* 2018;27(2):116-22. doi: 10.1177/0963721417738144
3. Gruber R, Wiebe ST, Wells SA, et al. Sleep and academic success: mechanisms, empirical evidence, and interventional strategies. *Adolescent medicine: state of the art reviews* 2010;21(3):522-41, x. [published Online First: 2011/02/10]
4. Perry GS, Patil SP, Presley-Cantrell LR. Raising awareness of sleep as a healthy behavior. *Prev Chronic Dis* 2013;10:E133-E33. doi: 10.5888/pcd10.130081
5. Walker M. Why we sleep: The new science of sleep and dreams. London: Penguin UK 2018.
6. Owens J. Insufficient Sleep in Adolescents and Young Adults: An Update on Causes and Consequences. *Pediatrics* 2014;134(3):E921-E32. doi: 10.1542/peds.2014-1696
7. Gradisar M, Wolfson AR, Harvey AG, et al. The sleep and technology use of Americans: findings from the National Sleep Foundation's 2011 Sleep in America poll. *Journal of clinical sleep medicine : JCSM : official publication of the American Academy of Sleep Medicine* 2013;9(12):1291-9. doi: 10.5664/jcsm.3272 [published Online First: 2013/12/18]
8. Eaton DK, McKnight-Eily LR, Lowry R, et al. Prevalence of Insufficient, Borderline, and Optimal Hours of Sleep Among High School Students – United States, 2007. *J Adolescent Health* 2010;46(4):399-401. doi: https://doi.org/10.1016/j.jadohealth.2009.10.011
9. Cain N, Gradisar M. Electronic media use and sleep in school-aged children and adolescents: A review. *Sleep Med* 2010;11(8):735-42. doi: 10.1016/j.sleep.2010.02.006
10. Vernon L, Barber BL, Modecki KL. Adolescent Problematic Social Networking and School Experiences: The Mediating Effects of Sleep Disruptions and Sleep Quality. *CyberPsychology, Behavior & Social Networking* 2015;18(7):386-92. doi: 10.1089/cyber.2015.0107
11. Przybylski AK. Digital Screen Time and Pediatric Sleep: Evidence from a Preregistered Cohort Study. *The Journal of Pediatrics* 2018 doi: 10.1016/j.jpeds.2018.09.054
12. Hale L, Guan S. Screen time and sleep among school-aged children and adolescents: a systematic literature review. *Sleep Med Rev* 2015;21:50-8. doi: 10.1016/j.smrv.2014.07.007 [published Online First: 2014/09/07]
13. Scott H, Biello S, Woods HC. Identifying drivers for bedtime social media use despite sleep costs: the adolescent perspective. . 12 Oct 2018 12 October 2018. https://doi.org/10.31234/osf.io/2xb36.
14. Scott H, Woods HC. Fear of missing out and sleep: Cognitive behavioural factors in adolescents' nighttime social media use. *Journal of Adolescence* 2018;68:61-65. doi: https://doi.org/10.1016/j.adolescence.2018.07.009
15. Arora T, Broglia E, Thomas GN, et al. Associations between specific technologies and adolescent sleep quantity, sleep quality, and parasomnias. *Sleep Med* 2014;15(2):240-47. doi: https://doi.org/10.1016/j.sleep.2013.08.799

For peer review only

16. Woods HC, Scott H. #Sleepyteens: Social media use in adolescence is associated with poor sleep quality, anxiety, depression and low self-esteem. *J Adolesc* 2016;51:41-9. doi: 10.1016/j.adolescence.2016.05.008
17. Levenson JC, Shensa A, Sidani JE, et al. Social media use before bed and sleep disturbance among young adults in the United States: a nationally representative study. *Sleep* 2017;40(9):zsx113-zsx13. doi: 10.1093/sleep/zsx113
18. Chang A-M, Aeschbach D, Duffy JF, et al. Evening use of light-emitting eReaders negatively affects sleep, circadian timing, and next-morning alertness. *Proceedings of the National Academy of Sciences* 2015;112(4):1232-37. doi: 10.1073/pnas.1418490112
19. Harbard E, Allen NB, Trinder J, et al. What's Keeping Teenagers Up? Prebedtime Behaviors and Actigraphy-Assessed Sleep Over School and Vacation. *J Adolescent Health* 2016;58(4):426-32. doi: 10.1016/j.jadohealth.2015.12.011
20. An J, Sun Y, Wan Y, et al. Associations between problematic internet use and adolescents' physical and psychological symptoms: possible role of sleep quality. *Journal of addiction medicine* 2014;8(4):282-7. doi: 10.1097/adm.0000000000000026 [published Online First: 2014/07/16]
21. Levenson JC, Shensa A, Sidani JE, et al. The association between social media use and sleep disturbance among young adults. *Prev Med* 2016;85:36-41. doi: 10.1016/j.ypmed.2016.01.001
22. Pew Research Center. Social Media Use in 2018, March 2018.
23. Centre for Longitudinal Studies. Millennium Cohort Study Sixth Sweep (MCS6): Technical Report: UCL Institute of Education, 2017.
24. Goodman R. Psychometric Properties of the Strengths and Difficulties Questionnaire. *Journal of the American Academy of Child & Adolescent Psychiatry* 2001;40(11):1337-45. doi: 10.1097/00004583-200111000-00015
25. Angold A, Costello EJ, Messer SC, et al. Development of a short questionnaire for use in epidemiological studies of depression in children and adolescents. *International Journal of Methods in Psychiatric Research* 1995;5(4):237-49.
26. Rosenberg M. Society and the adolescent self-image. 1965
27. Lichstein KL, Durrence HH, Taylor DJ, et al. Quantitative criteria for insomnia. *Behav Res Ther* 2003;41(4):427-45. doi: 10.1016/s0005-7967(02)00023-2
28. Espie CA, Kyle SD, Hames P, et al. The Sleep Condition Indicator: a clinical screening tool to evaluate insomnia disorder. *BMJ Open* 2014;4(3) doi: 10.1136/bmjopen-2013-004183
29. srvyr: 'dplyr'-like syntax for summary statistics of survey data. [program]. 0.3.0 version, 2018.
30. Lumley T. Analysis of Complex Survey Samples. *2004* 2004;9(8):19. doi: 10.18637/jss.v009.i08 [published Online First: 2004-01-08]
31. survey: analysis of complex survey samples [program]. 3.32 version, 2017.
32. Sterne JAC, White IR, Carlin JB, et al. Multiple imputation for missing data in epidemiological and clinical research: potential and pitfalls. *BMJ* 2009;338 doi: 10.1136/bmj.b2393
33. van Buuren S, Groothuis-Oudshoorn K. mice: Multivariate Imputation by Chained Equations in R. *J Stat Softw* 2011;45(3):1-67.

For peer review only

34. Zhang J, Yu KF. What's the relative risk? A method of correcting the odds ratio in cohort studies of common outcomes. *Jama* 1998;280(19):1690-1. [published Online First: 1998/12/01]
35. Pew Research Center. Teens' social media habits and experiences, November 2018.
36. Herring SC, Kapidzic S. Teens, Gender, and Self-Presentation in Social Media. In: Wright JD, ed. International Encyclopedia of the Social & Behavioral Sciences (Second Edition). Oxford: Elsevier 2015:146-52.
37. Galland BC, Short MA, Terrill P, et al. Establishing normal values for pediatric nighttime sleep measured by actigraphy: a systematic review and meta-analysis. *Sleep* 2018;41(4) doi: 10.1093/sleep/zsy017 [published Online First: 2018/03/29]
38. Hysing M, Pallesen S, Stormark KM, et al. Sleep patterns and insomnia among adolescents: a population-based study. *J Sleep Res* 2013;22(5):549-56. doi: 10.1111/jsr.12055 [published Online First: 2013/04/25]
39. Kuss DJ, Griffiths MD. Online Social Networking and Addiction—A Review of the Psychological Literature. *International Journal of Environmental Research and Public Health* 2011;8(9) doi: 10.3390/ijerph8093528
40. Przybylski AK, Weinstein N. A Large-Scale Test of the Goldilocks Hypothesis: Quantifying the Relations Between Digital-Screen Use and the Mental Well-Being of Adolescents. *Psychological Science* 2017;28(2):204-15. doi: 10.1177/0956797616678438
41. Asarnow LD, McGlinchey E, Harvey AG. The effects of bedtime and sleep duration on academic and emotional outcomes in a nationally representative sample of adolescents. *The Journal of adolescent health : official publication of the Society for Adolescent Medicine* 2014;54(3):350-6. doi: 10.1016/j.jadohealth.2013.09.004 [published Online First: 2013/11/15]
42. Exelmans L, Van den Bulck J. Bedtime, shuteye time and electronic media: sleep displacement is a two-step process. *J Sleep Res* 2017;26(3):364-70. doi: 10.1111/jsr.12510
43. Carskadon MA. Sleep in adolescents: the perfect storm. *Pediatric clinics of North America* 2011;58(3):637-47. doi: 10.1016/j.pcl.2011.03.003
44. Roenneberg T, Kuehnle T, Pramstaller PP, et al. A marker for the end of adolescence. *Current Biology* 2004;14(24):R1038-R39. doi: http://dx.doi.org/10.1016/j.cub.2004.11.039
45. Owens J, Drobnich D, Baylor A, et al. School Start Time Change: An In-Depth Examination of School Districts in the United States. *Mind, Brain, and Education* 2014;8(4):182-213. doi: 10.1111/mbe.12059
46. Espinoza G, Juvonen J. The Pervasiveness, Connectedness, and Intrusiveness of Social Network Site Use Among Young Adolescents. *Cyberpsych Beh Soc N* 2011;14(12):705-09. doi: 10.1089/cyber.2010.0492
47. Davis K. Friendship 2.0: adolescents' experiences of belonging and self-disclosure online. *J Adolesc* 2012;35(6):1527-36. doi: 10.1016/j.adolescence.2012.02.013
48. Davis K. Young people's digital lives: The impact of interpersonal relationships and digital media use on adolescents' sense of identity. *Computers in Human Behavior* 2013;29(6):2281-93. doi: https://doi.org/10.1016/j.chb.2013.05.022
49. Reynolds CF, 3rd, O'Hara R. DSM-5 sleep-wake disorders classification: overview for use in clinical practice. *The American journal of psychiatry* 2013;170(10):1099-101. doi: 10.1176/appi.ajp.2013.13010058 [published Online First: 2013/10/03]

For peer review only

50. Przybylski AK, Murayama K, DeHaan CR, et al. Motivational, emotional, and behavioral correlates of fear of missing out. *Computers in Human Behavior* 2013;29(4):1841-48. doi: 10.1016/j.chb.2013.02.014
51. Fox J, Moreland JJ. The dark side of social networking sites: An exploration of the relational and psychological stressors associated with Facebook use and affordances. *Computers in Human Behavior* 2015;45:168-76. doi: 10.1016/j.chb.2014.11.083
52. Tubbs AS, Dollish HK, Fernandez F, et al. Chapter 1 - The basics of sleep physiology and behavior. In: Grandner MA, ed. Sleep and Health: Academic Press 2019:3-10.
53. Fernandez-Mendoza J, Calhoun SL, Bixler EO, et al. Sleep misperception and chronic insomnia in the general population: role of objective sleep duration and psychological profiles. *Psychosomatic medicine* 2011;73(1):88-97. doi: 10.1097/PSY.0b013e3181fe365a [published Online First: 2010/10/28]
54. Silva GE, Goodwin JL, Sherrill DL, et al. Relationship between reported and measured sleep times: the sleep heart health study (SHHS). *Journal of clinical sleep medicine : JCSM : official publication of the American Academy of Sleep Medicine* 2007;3(6):622-30.
55. Gregory AM, Sadeh A. Sleep, emotional and behavioral difficulties in children and adolescents. *Sleep Med Rev* 2012;16(2):129-36. doi: https://doi.org/10.1016/j.smrv.2011.03.007
56. Bartel K, Gradisar M. New Directions in the Link Between Technology Use and Sleep in Young People. In: Nevšímalová S, Bruni O, eds. Sleep Disorders in Children. Cham: Springer International Publishing 2017:69-80.
57. Bhat S, Pinto-Zipp G, Upadhyay H, et al. "To sleep, perchance to tweet": in-bed electronic social media use and its associations with insomnia, daytime sleepiness, mood, and sleep duration in adults. *Sleep Health* 2018;4(2):166-73. doi: https://doi.org/10.1016/j.sleh.2017.12.004
58. Mazzer K, Bauducco S, Linton SJ, et al. Longitudinal associations between time spent using technology and sleep duration among adolescents. *J Adolesc* 2018;66:112-19. doi: 10.1016/j.adolescence.2018.05.004 [published Online First: 2018/05/31]
59. Bartel K, Scheeren R, Gradisar M. Altering Adolescents' Pre-Bedtime Phone Use to Achieve Better Sleep Health. *Health Communication* 2018:1-7. doi: 10.1080/10410236.2017.1422099

For peer review only

Supplementary Materials

Full breakdown of sleep patterns

Supplementary Table 1. Typical sleep onset (school days) for males & females

Typical sleep onset on school days	Male		Female		Total	
	%	n	%	n	%	n
Before 9 pm	5.8	288	4.7	248	5.2	536
9 - 9:59 pm	28.5	1619	30.0	1681	29.2	3300
10 - 10:59 pm	40.2	2352	38.8	2284	39.5	4636
11 pm - midnight	18.7	1076	20.3	1220	19.5	2296
After midnight	6.9	366	6.1	335	6.5	701

Notes: percentages account for survey design and weights.

Supplementary Table 2. Typical sleep onset (free days) for males & females

Typical sleep onset on free days	Male		Female		Total	
	%	n	%	n	%	n
Before 9 pm	0.9	53	0.9	45	0.9	98
9 - 9:59 pm	6.0	328	5.4	299	5.7	627
10 - 10:59 pm	22.8	1311	24.4	1407	23.6	2718
11 pm - midnight	35.1	2044	37.2	2208	36.1	4252
After midnight	35.2	1969	32.1	1808	33.7	3777

Notes: percentages account for survey design and weights. Gender difference in sleep onset (free days) $p < .05$.

Supplementary Table 3. Typical wake time (school days) for males & females

Typical wake time on school days	Male		Female		Total	
	%	n	%	n	%	n
Before 6 am	4.4	220	4.5	230	4.5	450
6 - 6:59 am	37.0	1995	47.9	2609	42.2	4604
7 - 7:59 am	53.3	3205	44.9	2771	49.3	5976
8 - 8:59 am	4.0	247	2.1	130	3.1	377
After 9 am	1.3	42	0.6	32	1.0	74

Notes: percentages account for survey design and weights. Gender difference in wake time (school days) $p < .001$.

For peer review only

Supplementary Table 4. Typical wake time (free days) for males & females

Typical wake time on free days	Male		Female		Total	
	%	n	%	n	%	n
Before 8 am	9.9	545	6.4	342	8.2	887
8 - 8:59 am	17.1	940	15.6	846	16.3	1786
9 - 9:59 am	22.9	1348	26.5	1537	24.6	2885
10 - 10:59 am	27.6	1628	30.2	1763	28.8	3391
11 - 11:59 am	14.8	836	15.3	934	15.0	1770
After midday	7.8	401	6.2	348	7.0	749

Notes: percentages account for survey design and weights. Gender difference in wake time (free days) $p < .001$.

Supplementary Table 5. Typical sleep onset latency for males & females

Typical sleep onset latency	Male		Female		Total	
	%	n	%	n	%	n
0 - 15 minutes	37.1	2104	29.3	1709	33.3	3813
16 - 30 minutes	31.4	1838	33.7	1963	32.6	3801
31 - 45 minutes	14.8	819	17.3	973	16.0	1792
46 - 60 minutes	6.4	383	8.7	495	7.5	878
More than 60 minutes	10.3	516	11.0	610	10.6	1126

Notes: percentages account for survey design and weights. Gender difference in sleep onset latency $p < .001$.

Supplementary Table 6. Frequency of nighttime awakenings for males & females

Frequency of nighttime awakenings	Male		Female		Total	
	%	n	%	n	%	n
None of the time	35.8	2050	24.1	1435	30.1	3485
A little of the time	32.8	1951	32.7	1966	32.8	3917
Some of the time	14.5	831	17.6	991	16.0	1822
A good bit of the time	7.0	380	10.7	583	8.8	963
Most of the time	6.7	337	10.7	554	8.6	891
All of the time	3.1	143	4.3	226	3.7	369

Notes: percentages account for survey design and weights. Gender difference in nighttime awakenings $p < .001$.

For peer review only

Social media use by demographics

Table 1 in the main text provides a breakdown of social media use by gender. Supplementary tables 7 and 8 provide a breakdown of social media use by additional demographics: household income and ethnic minority status.

Supplementary Table 7. Social media use by household equivalised income

		Social media use: Low <1 h	Average 1 to <3 h	High 3 to <5 h	Very high 5+ h	Total n
**Household equivalized income**	Quintile 1 (lowest)	33.9	28.7	11.3	26.1	2038
	Quintile 2	30.6	29.2	16.3	23.9	2008
	Quintile 3	31.6	30.3	15.2	22.9	2414
	Quintile 4	32.4	35.8	14.0	17.9	2727
	Quintile 5 (highest)	40.0	33.8	12.7	13.6	2685

Notes: Numbers represent row percentages (e.g. 33.9% of cohort members in lowest income quintile were low social media users). Percentages account for survey design and weights. Income difference in social media use $p < .001$.

Supplementary Table 8. Social media use by ethnicity

		Social media use: Low <1 h	Average 1 to <3 h	High 3 to <5 h	Very high 5+ h	Total n
**Ethnicity**	White	32.9	31.7	14.4	21.1	9086
	Non-white	37.3	31.4	11.9	19.4	2326

Notes: Numbers represent row percentages (e.g. 32.9% of White cohort members were low social media users). Percentages account for survey design and weights. Ethnicity difference in social media use $p < .05$.

For peer review only

Survey questions

About what time do you usually **go to sleep** on a **school night**?
1 Before 9 pm
2 9 - 9:59 pm
3 10 – 10:59 pm
4 11 - midnight
5 After midnight

About what time do you usually **wake up** in the morning on a **school day**?
1 Before 6 am
2 6 - 6:59 am
3 7 – 7:59 am
4 8 - 8:59 am
5 After 9 am

About what time do you usually **go to sleep** on the nights when you **do not have school** the next day?
1 Before 9 pm
2 9 - 9:59 pm
3 10 - 10:59 pm
4 11 - midnight
5 After midnight

About what time do you **wake up** in the morning on the days when you **do not have school**?
1 Before 8 am
2 8 - 8:59 am
3 9 – 9:59 am
4 10 - 10:59 am
5 11 - 11:59 am
6 After Midday

During the last four weeks, how long did it usually take for you to fall asleep?
1 0-15 minutes
2 16-30 minutes
3 31-45 minutes
4 46-60 minutes
5 More than 60 minutes

During the last four weeks, how often did you awaken during your sleep time and have trouble falling back to sleep again?
1 All of the time
2 Most of the time
3 A good bit of the time
4 Some of the time
5 A little of the time
6 None of the time

On a normal week day during term time, how many hours do you spend on social networking or messaging sites or Apps on the internet such as Facebook, Twitter and WhatsApp?
1 None
2 Less than half an hour
3 Half an hour to less than 1 hour
4 1 hour to less than 2 hours
5 2 hours to less than 3 hours
6 3 hours to less than 5 hours

For peer review only

7 5 hours to less than 7 hours
8 7 hours or more

For peer review only

Figures

Figure 1. Odds Ratios of sleep outcomes for low, high & very high daily social media users

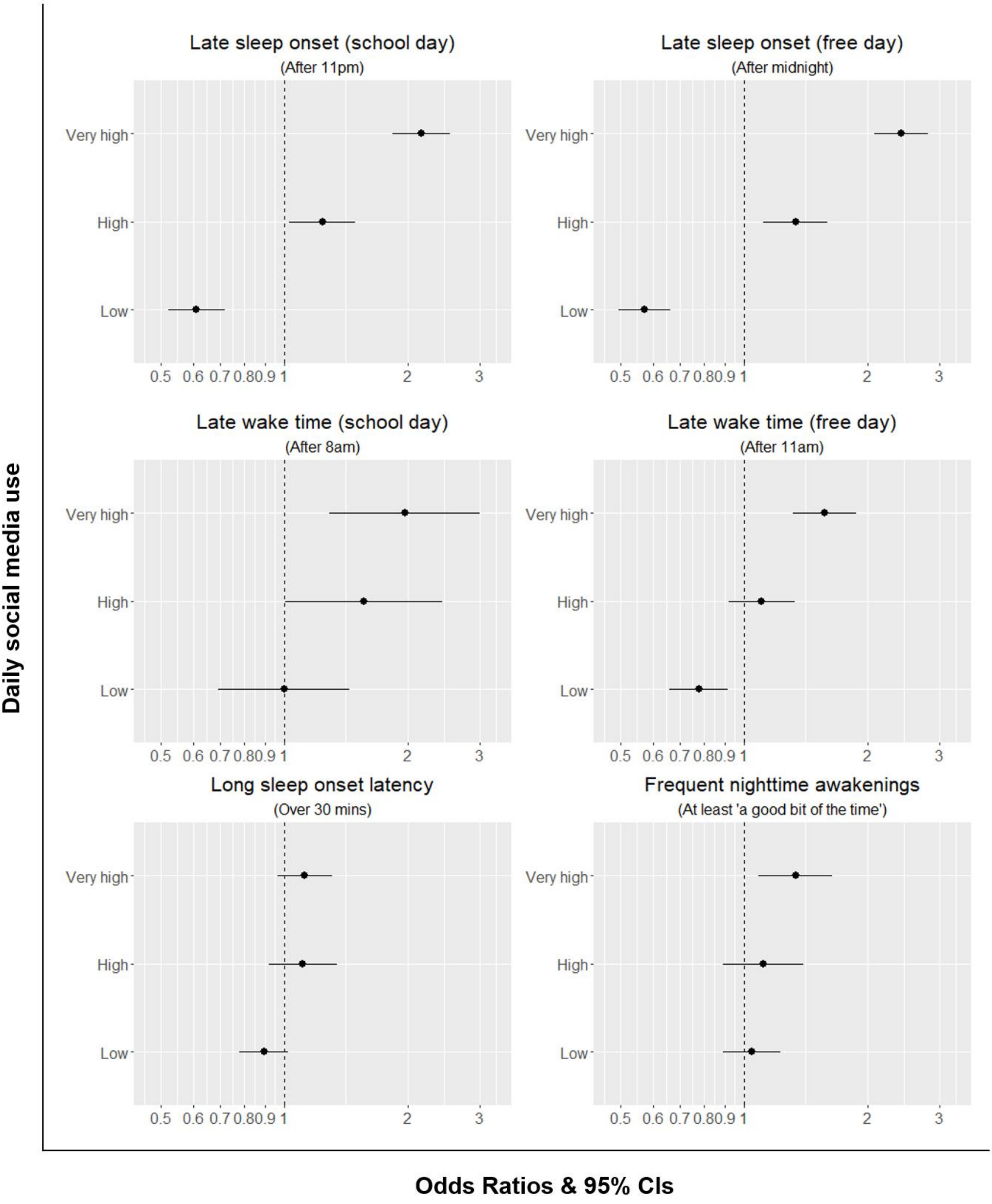

Notes: Odds Ratios measure how much higher or lower the odds of a given sleep outcome are for each category of social media user (Low, <1h; High, 3-5h; Very high, 5+h) compared to typical users (1-3h). Odds Ratios and 95% Confidence Intervals greater than 1 indicate higher odds; those below 1 indicate lower odds. Odds Ratios are adjusted to control for: exact age, sex, ethnic minority status, family income, number of siblings in household, presence of both parents, parent age, Strengths and Difficulties score, Mood and Feelings score, self-esteem, general health, social support and physical activity.

STROBE Statement—Checklist of items that should be included in reports of ***cross-sectional studies***

	Item No	Recommendation	Page No
**Title and abstract**	1	(*a*) Indicate the study's design with a commonly used term in the title or the abstract	1
		(*b*) Provide in the abstract an informative and balanced summary of what was done and what was found	2
**Introduction**			
Background/rationale	2	Explain the scientific background and rationale for the investigation being reported	4-6
Objectives	3	State specific objectives, including any prespecified hypotheses	5-6
**Methods**			
Study design	4	Present key elements of study design early in the paper	7
Setting	5	Describe the setting, locations, and relevant dates, including periods of recruitment, exposure, follow-up, and data collection	7
Participants	6	(*a*) Give the eligibility criteria, and the sources and methods of selection of participants	7
Variables	7	Clearly define all outcomes, exposures, predictors, potential confounders, and effect modifiers. Give diagnostic criteria, if applicable	7-8
Data sources/ measurement	8*	For each variable of interest, give sources of data and details of methods of assessment (measurement). Describe comparability of assessment methods if there is more than one group	7-8
Bias	9	Describe any efforts to address potential sources of bias	NA
Study size	10	Explain how the study size was arrived at	7
Quantitative variables	11	Explain how quantitative variables were handled in the analyses. If applicable, describe which groupings were chosen and why	8
Statistical methods	12	(*a*) Describe all statistical methods, including those used to control for confounding	9
		(*b*) Describe any methods used to examine subgroups and interactions	NA
		(*c*) Explain how missing data were addressed	9
		(*d*) If applicable, describe analytical methods taking account of sampling strategy	9
		(*e*) Describe any sensitivity analyses	9
**Results**			
Participants	13*	(a) Report numbers of individuals at each stage of study—eg numbers potentially eligible, examined for eligibility, confirmed eligible, included in the study, completing follow-up, and analysed	7
		(b) Give reasons for non-participation at each stage	NA
		(c) Consider use of a flow diagram	NA
Descriptive data	14*	(a) Give characteristics of study participants (eg demographic, clinical, social) and information on exposures and potential confounders	8-9
		(b) Indicate number of participants with missing data for each variable of interest	10
Outcome data	15*	Report numbers of outcome events or summary measures	8-9
Main results	16	(*a*) Give unadjusted estimates and, if applicable, confounder-adjusted estimates and their precision (eg, 95% confidence interval). Make clear which confounders were adjusted for and why they were included	10-12

For peer review only

		(*b*) Report category boundaries when continuous variables were categorized	8
		(*c*) If relevant, consider translating estimates of relative risk into absolute risk for a meaningful time period	NA
Other analyses	17	Report other analyses done—eg analyses of subgroups and interactions, and sensitivity analyses	NA
**Discussion**			
Key results	18	Summarise key results with reference to study objectives	13
Limitations	19	Discuss limitations of the study, taking into account sources of potential bias or imprecision. Discuss both direction and magnitude of any potential bias	15-16
Interpretation	20	Give a cautious overall interpretation of results considering objectives, limitations, multiplicity of analyses, results from similar studies, and other relevant evidence	14-15
Generalisability	21	Discuss the generalisability (external validity) of the study results	16
**Other information**			
Funding	22	Give the source of funding and the role of the funders for the present study and, if applicable, for the original study on which the present article is based	17

*Give information separately for exposed and unexposed groups.

Note: An Explanation and Elaboration article discusses each checklist item and gives methodological background and published examples of transparent reporting. The STROBE checklist is best used in conjunction with this article (freely available on the Web sites of PLoS Medicine at http://www.plosmedicine.org/, Annals of Internal Medicine at http://www.annals.org/, and Epidemiology at http://www.epidem.com/). Information on the STROBE Initiative is available at www.strobe-statement.org.

For peer review only

For peer review only - http://bmjopen.bmj.com/site/about/guidelines.xhtml
